# PP1γ regulates neuronal insulin signaling and aggravates insulin resistance leading to AD-like phenotypes

**DOI:** 10.1186/s12964-023-01071-x

**Published:** 2023-04-21

**Authors:** Yamini Yadav, Medha Sharma, Chinmoy Sankar Dey

**Affiliations:** Kusuma School of Biological Sciences, Indian Institute of Technology, Delhi, New Delhi 110016 India

**Keywords:** PP1γ, AKT isoforms, MLK3, Neuronal insulin signaling, Insulin resistance, Alzheimer disease

## Abstract

**Background:**

PP1γ is one of the isoforms of catalytic subunit of a Ser/Thr phosphatase PP1. The role of PP1γ in cellular regulation is largely unknown. The present study investigated the role of PP1γ in regulating neuronal insulin signaling and insulin resistance in neuronal cells. PP1 was inhibited in mouse neuroblastoma cells (N2a) and human neuroblastoma cells (SH-SY5Y). The expression of PP1α and PP1γ was determined in insulin resistant N2a, SH-SY5Y cells and in high-fat-diet-fed-diabetic mice whole-brain-lysates. PP1α and PP1γ were silenced by siRNA in N2a and SH-SY5Y cells and effect was tested on AKT isoforms, AS160 and GSK3 isoforms using western immunoblot, GLUT4 translocation by confocal microscopy and glucose uptake by fluorescence-based assay.

**Results:**

Results showed that, in one hand PP1γ, and not PP1α, regulates neuronal insulin signaling and insulin resistance by regulating phosphorylation of AKT2 via AKT2-AS160-GLUT4 axis. On the other hand, PP1γ regulates phosphorylation of GSK3β via AKT2 while phosphorylation of GSK3α via MLK3. Imbalance in this regulation results into AD-like phenotype.

**Conclusion:**

PP1γ acts as a linker, regulating two pathophysiological conditions, neuronal insulin resistance and AD.

Video Abstract

**Supplementary Information:**

The online version contains supplementary material available at 10.1186/s12964-023-01071-x.

## Introduction

Neuronal insulin signaling regulates myriads of processes like neuronal survival, neurotransmission, and cognitive performance [1]. Any impairment in insulin signaling leads to insulin resistance, which has been linked to various neurodegenerative disorders like Alzheimer’s disease (AD) [2], Parkinson’s disease (PD) [3] Huntington’s disease (HD) [4] etc. In contrast to peripheral tissues like skeletal muscle, liver and adipocytes where insulin signaling and effect of insulin resistance has been studied extensively, the regulation of neuronal insulin signaling is not explored as much. Phosphorylation/dephosphorylation of important proteins in insulin signaling cascade is one of the key regulatory events. To fully understand regulation of neuronal insulin signaling, unraveling the processes regulated through complementary and countervailing actions of protein kinases and phosphatases are extremely crucial. So far, the contributions of kinases like AKT [5], CDK5 [6], PKC [7] and PAK2 [8] have been studied addressing insulin signaling and insulin resistance in neuronal system. Conversely, studies reporting the role of phosphatases is anyway very limited, with an even lesser number of studies in the neuronal system.

One such phosphatase is Protein Phosphatase 1 (PP1). PP1 belongs to Ser/Thr phosphatase, a heteromeric protein composed of three isoforms of catalytic subunits naming PP1α, PP1β/δ and PP1γ interacting with > 200 regulatory subunits. The ability of PP1 to recognize different substrates lies in the combination of binding of different isoforms of catalytic subunit(s) with different regulatory subunit(s). Previously, some studies have reported the role of PP1 in insulin signaling. Corvera et al. [9] and Rondinone et al. [10] suggested the participation of a phosphatase responsible for GLUT4 transport in response to insulin in adipocyte. A small number of studies demonstrate specific role of catalytic and regulatory subunits of PP1, acting on different molecules of insulin signaling cascade. Geetha et al. [11] observed that in skeletal muscle, catalytic subunit PP1δ combines with PP1 regulatory subunit PP1R12A, and acts on IRS-1, regulating inulin signaling cascade. Sharma et al. [12] demonstrated that PP1α catalytic subunit acts directly on AS160, dephosphorylate it and regulate insulin signaling in skeletal muscle.

PP1 and isoforms of catalytic subunit are abundantly expressed in mammalian brain. Among three isoforms of catalytic subunits of PP1, PP1α and PP1γ, are reported to be highly expressed in brain [13]. However, role of PP1 and isoforms of PP1 catalytic subunits is not yet studied in neuronal insulin signaling and resistance. Innumerable reports connected neuronal insulin resistance to AD [14–17]. PP1 has been linked to AD pathogenesis [18]. However, possible role of PP1 isoforms in regulating both pathological disorders, if any, has not been reported.

In our current study, we for the first time determined regulatory role of PP1, and more precisely PP1γ, in neuronal insulin signaling and insulin resistance. We also found that PP1γ is involved in precipitating AD-like phenotypes. PP1γ acts, possibly, like a linker in the progression of insulin resistance and AD-like pathogenesis. Widespread studies in future are required for in depth understanding to recognize the role of PP1γ in the development of AD pathogenesis in diabetic conditions.

## Results

### Effect of PP1 inhibition on neuronal insulin signaling

**Fig. 1:**
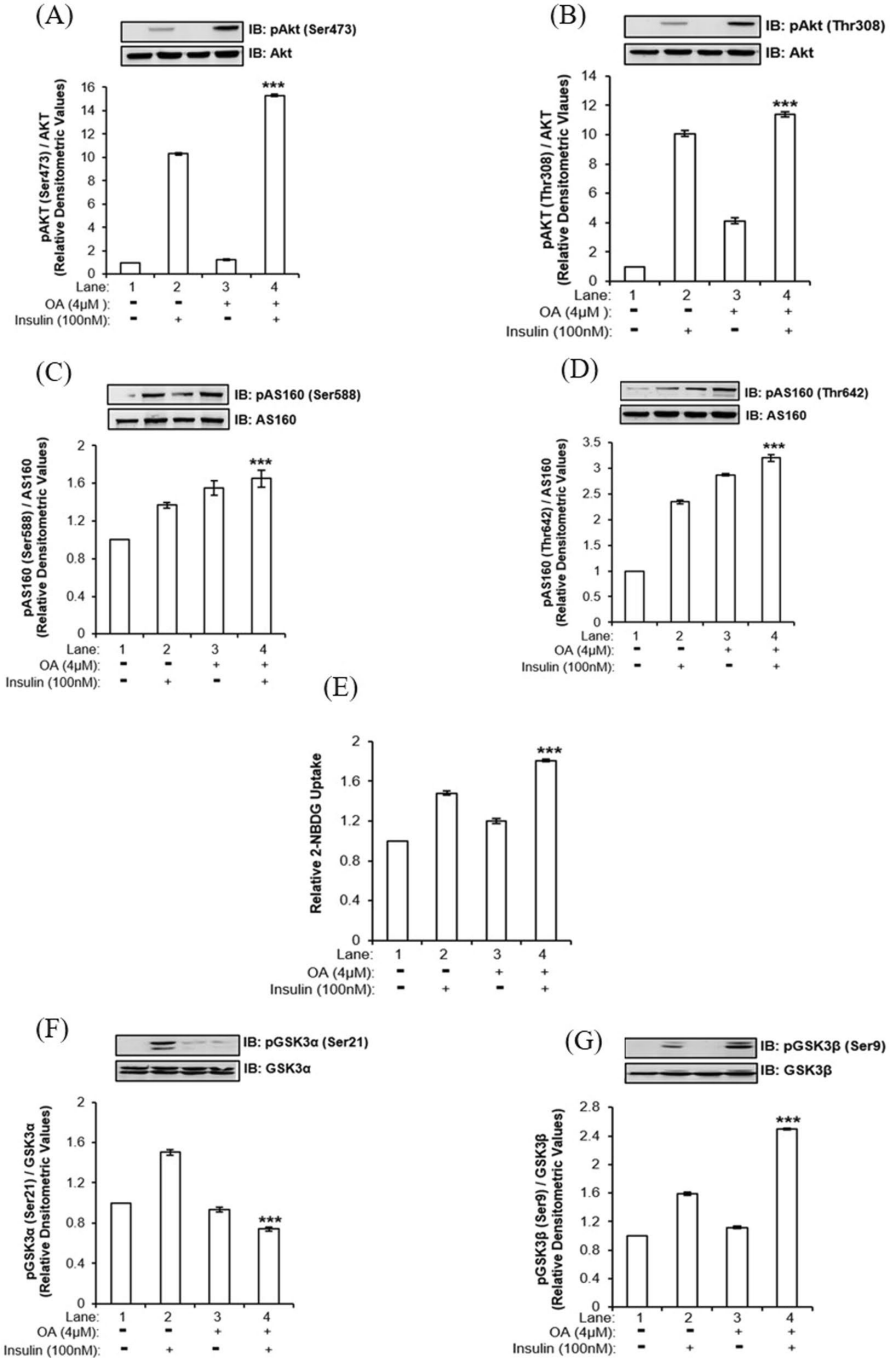
Effect of PP1 inhibition on neuronal insulin signaling in N2a cells: Differentiated N2a cells were treated with or without 4 μM OA for 120 min, followed by 100 nM insulin for 30 min. Treated cells were lysed and subjected to western immunoblotting followed by probing with relevant primary antibodies. Bar represents relative change in **A** pAKT (Ser473) **B** pAKT (Thr308) **C** pAS160 (Ser588) **D** pAS160 (Thr642) **F** pGSK3α (Ser21) **G** pGSK3β (Ser9). For glucose uptake assay differentiated N2a cells were serum-starved for 2 h and then treated with or without 4 μM OA for 120 min, followed by 100 nM insulin for 30 min. Uptake of 2-NBDG was then measured. Bar represents (**E**) relative change in the uptake of 2-NBDG. Experiments were executed three times and a representative western blot is shown. Data expressed are mean ± SE ***p < 0.001 compared to Lane 2. (**A** and **B**) AKT was used as a loading control (**C** and **D**) AS160 was used as a loading control (**F**) pGSK3α was used as a loading control (**G**) pGSK3β was used as a loading control. *A.U.* Arbitrary Units. *IB* Immunoblot, *OA* Okadaic Acid

To understand the role of PP1, if any, in neuronal insulin signaling, we inhibited PP1 by Okadaic Acid (OA) [19] and tested the effect of inhibition on activation of AKT (as determined by phosphorylation at Ser473 and Thr308). Differentiated N2a cells were treated with or without 4 μM OA for 120 min followed by 100 nM insulin for 30 min [19]. Treated cells were lysed and protein lysates were subjected to western immunoblotting and probed with specific antibodies. Cells treated with OA showed 49 ± 0.06% and 34 ± 0.10% increase in phosphorylation of AKT at Ser473 and Thr308 respectively (Fig. 1A and B, Lane 4 vs. Lane 2, ***p < 0.001) in insulin stimulated N2a cells. This provided us with the first evidence that PP1 is involved in neuronal insulin signaling. Since PP1 inhibition affected the activation of AKT, we next tested the effect of PP1 inhibition on activation of immediate downstream target of AKT i.e., AS160. AS160 is a Rab-GTPase that regulates translocation of GLUT4 from the cytoplasm to the plasma membrane in response to insulin, leading to glucose uptake. Post insulin stimulation, activation of AS160 (as determined by phosphorylation at Ser588 and Thr642) was found to be increased by 37 ± 0.03% and 46 ± 0.02% respectively post PP1 inhibition (Fig. 1C and D, Lane 4 vs. Lane 2, ***p < 0.001). Having observed PP1 inhibition regulated the activities of AKT and AS160, we next tested the effect of PP1 inhibition on neuronal glucose uptake. Cells treated with 4 μM OA for 120 min followed by 100 nM insulin for 30 min were subjected to 2-NBDG uptake [6, 7, 5–22], displayed 22 ± 0.01% increase in neuronal glucose uptake as compared to control (Fig. 1E, Lane 4 vs. Lane 2, ***p < 0.001).

We next tested the effect of PP1 inhibition on activation of another AKT downstream substrate, GSK3. GSK3 has two isoforms, GSK3α and GSK3β, which when undergoes phosphorylation at Ser21 and Ser9 residues respectively, gets inactivated. We observed that post PP1 inhibition, phosphorylation of GSK3α at Ser21 was decreased by 51 ± 0.02% (Fig. 1F, Lane 4 vs. Lane 2, ***p < 0.001). On the contrary, phosphorylation of GSK3β was increased by 57 ± 0.01% (Fig. 1G, Lane 4 vs. Lane 2, ***p < 0.001) in response to insulin.

To determine whether the above-mentioned effects were not cell and species specific, we tested the effect of PP1 inhibition on the activations of AKT, AS160, GSK3 and uptake of glucose in differentiated human neuroblastoma SH-SY5Y cells. We observed results similar to N2a cells, that is, as a function of PP1 inhibition, phosphorylation of AKT and AS160, neuronal glucose uptake was increased post insulin stimulation and phosphorylations of GSK3 isoforms were affected oppositely (Additional file 1: Fig. S1). Data suggests that PP1 participates in neuronal insulin signaling by dephosphorylating AKT and regulating the phosphorylations of two GSK3 isoforms.

### Effect of insulin stimulation on expression of PP1α and PP1γ in insulin sensitive and insulin resistant neuronal cells

**Fig. 2:**
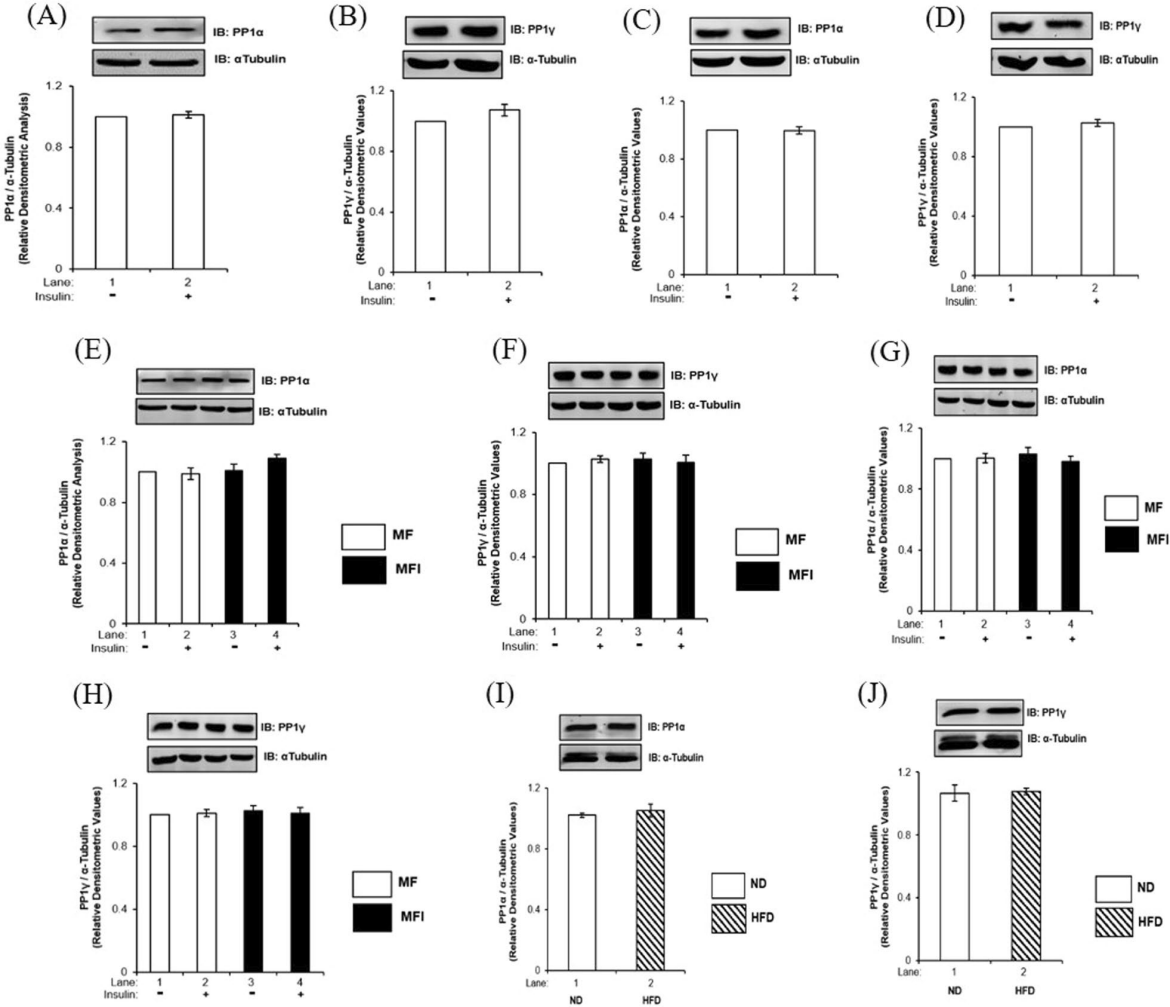
Effect of insulin stimulation on expression of PP1α and PP1γ in insulin sensitive and insulin resistant condition: Differentiated **A** and **B** N2a cells **C** and **D** SH-SY5Y cells were treated with or without 100 nM insulin for 30 min and lysed **E** and **F** ND and HFD mice brains were lysed **G** and **H** N2a cells were differentiated in insulin sensitive (MF) or in insulin resistant (MFI) conditions for 3 days and treated with or without 100 nM insulin for 30 min and were lysed, **I** and **J** SH-SY5Y cells were differentiated in insulin sensitive (MF) or in insulin resistant (MFI) conditions for 4 days and were treated with or without 100 nM insulin for 30 min and lysed. Resulting lysates were subjected to western immunoblotting and probed with relevant antibodies Bar represents relative change in expression of PP1α (**A**, **C**, **E**, **G** and I) and PP1γ (**B**, **D**, **F**, **H** and **J**). Experiments were executed three times and representative western blot is shown. Data expressed are mean ± SE. Open bars: MF, filled bars: MFI, *IB* Immunoblot, *ND* Normal diet, *HFD* High-fat-fed-diet. α-Tubulin was used as loading control

Among the three isoforms, namely PP1α, PP1β/δ and PP1γ, PP1α and PP1γ are reported to be predominantly expressed in mammalian brain [13]. Therefore, in order to figure out which isoform(s) is/are participate in the neuronal insulin signaling, we first tested the expression of PP1α and PP1γ in neuronal insulin sensitive and insulin resistant N2a and SH-SY5Y cells. To generate insulin resistant neuronal cells, we have subjected the cells in hyperinsulinemic condition, as reported previously from our laboratory [6, 7, 5–22]. As described in “materials and methods” section, we differentiated N2a and SH-SY5Y cells in a serum free insulin sensitive (MF) condition and an insulin resistant (MFI) condition in the chronic presence of 100 nM insulin for three days and then the cells were stimulated with or without 100 nM insulin for 30 min, subjected to western immunoblotting, probed with PP1α and PP1γ specific antibodies. We observed that both PP1α and PP1γ isoforms are expressed in N2a and SH-SY5Y cells under insulin sensitive and resistant conditions and insulin did not alter the expression of neither of the isoforms under any conditions tested (Fig. 2A–H). We also tested the expression of PP1α and PP1γ in whole mice brain lysates of High-fat-diet (HFD) fed insulin resistant mice and Normal-diet (ND) fed mice. No change in expression of PP1α and PP1γ was observed in HFD mice whole brain lysates when compared to ND (Fig. 2I and J). Data shows expression of PP1α and PP1γ in insulin sensitive and insulin resistant neuronal cells.

### Effect of PP1α and PP1γ silencing on AKT isoforms and AS160 in insulin sensitive and insulin resistant neuronal cells

Having observed the expression of both PP1α and PP1γ in insulin sensitive and insulin resistant neuronal cells we proceeded to further investigate the role of each isoform of PP1 catalytic subunits in regulating downstream substrates. To test the effect, isoforms were individually silenced and the effect on expression and activation were tested on the downstream substrates, with or without insulin stimulation. Silencing was optimized at 100 nM of siRNA for PP1α (Additional file 1: Fig S2A) and PP1γ, each (Additional file 1: Fig S2B). Cells were transfected with 100 nM of either PP1α or PP1γ siRNA and then subjected to differentiation under MF or MFI condition, with or without insulin stimulation. Lysates were subjected to western immunoblotting probed with relevant antibodies as and when mentioned with reference to the relevant experiment.

**Fig. 3:**
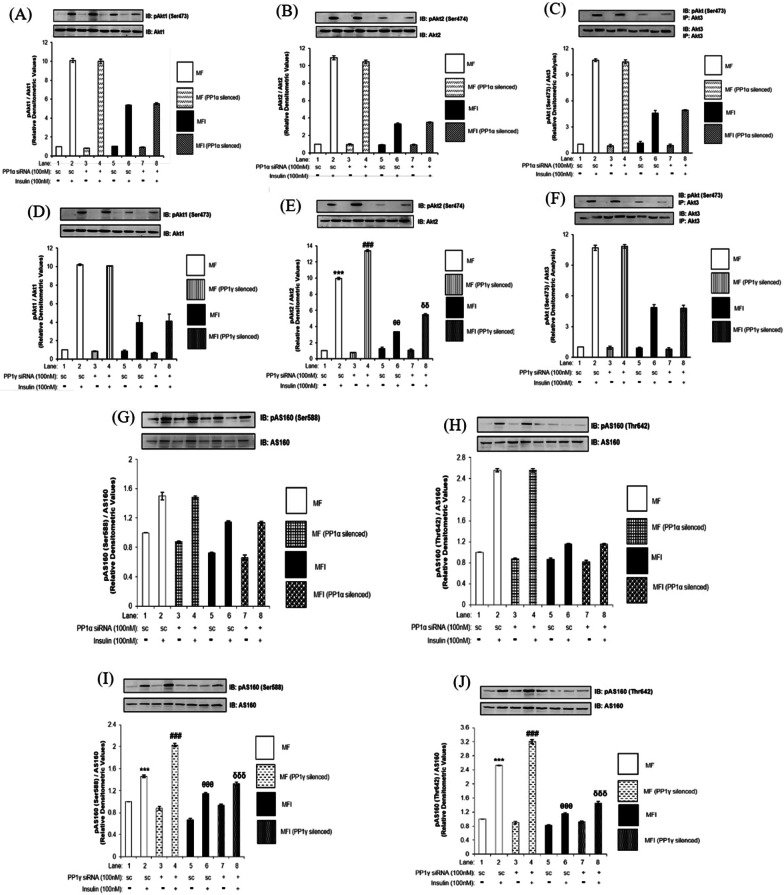
Effect of PP1α and PP1γ silencing on AKT isoforms and AS160 in insulin sensitive and insulin resistant condition: Proliferated N2a cells were transfected with non-specific (scrambled) and PP1α and PP1γ specific siRNA. Post transfection cells were differentiated in the absence (MF; insulin sensitive) or chronic presence of 100 nM insulin (MFI; insulin resistant) for 3 days. Transfected N2a cells were treated with or without 100 nM insulin for 30 min, lysed and probed with relevant primary antibodies for immunoblotting. **C** and **F** Post insulin stimulation, lysates were subjected to immunoprecipitation using anti-Akt3 antibody. Bar represents relative change in **A** and **D** pAKT1 (Ser473) **B** and **E** pAKT2 (Ser474) **C** and **F** pAKT (Ser473) **G** and **H** pAS160 (Ser588) **I** and **J** pAS160 (Thr672). Experiments were executed three times and a representative western blot is shown. Data expressed are mean ± SE. ***p < 0.001 compared to Lane 1, ^###^*p* < 0.001 compared to Lane 2, ^θθθ^*p* < 0.001 compared to lane 4 and ^δδδ^*p* < 0.001 compared to Lane 6. (**A** and **D**) AKT1, (**B** and **E**) AKT2, (**C** and **F**) AKT3, (**G**–**J**) AS160 was used as a loading control. Open bars: MF, filled bars: MFI, *IP* Immunoprecipitation *IB* Immunoblot, *SC* Scrambled

PP1α silencing did not affect the expression or phosphorylation of any isoform of AKT both under insulin sensitive (MF) and insulin resistant (MFI) condition when compared to respective control (Fig. 3A–C, Lane 4 vs. Lane 2 and Lane 8 vs. Lane 6). However, PP1γ silencing affected the phosphorylation of only AKT2 (Fig. 3E), while phosphorylation of AKT1 (Fig. 3D) and AKT3 (Fig. 3F) remained unaffected. PP1γ silenced cells under insulin sensitive condition showed 36 ± 0.1% increase in phosphorylation of AKT2 and 64 ± 0.15% increase in phosphorylation of AKT2 under insulin resistant condition as compared to control (Fig. 3E, Lane 4 vs. Lane 2, ^###^*p* < 0.001 and Lane 8 vs. Lane 6 respectively, ^δδ^*p* < 0.01). When phosphorylation of downstream target of AKT i.e., was tested, the phosphorylation of AS160 was unaffected both at Ser588 and Thr642 residues post PP1α silencing under insulin sensitive and insulin resistant condition when compared to control (Fig. 3G and H). However, cells transfected with PP1γ siRNA showed 39 ± 0.02% (Fig. 3I, Lane 4 vs. Lane 2, ^###^*p* < 0.001) and 16 ± 0.02% (Fig. 3I, Lane 6 vs. Lane 4, ^δδδ^*p* < 0.001) increase in phosphorylation of AS160 at Ser588 under insulin sensitive and insulin resistant condition, respectively, when compared to cells transfected with scrambled siRNA. Phosphorylation of AS160 at Thr642 was also found to be increased by 27 ± 0.05% (Fig. 3J, Lane 4 vs. Lane 2, ^###^*p* < 0.001) and 77 ± 0.04% (Fig. 3J, Lane 8 vs. Lane 6, ^δδδ^*p* < 0.001) under insulin sensitive and insulin resistant condition, respectively, when compared to control. Data shows that PP1α does not regulate any of the three AKT isoforms in neuronal insulin signaling and insulin resistance. In contrast, PP1γ regulates phosphorylation of AKT2 only, without affecting the phosphorylation of AKT1 or AKT3, in turn affecting the phosphorylation of AS160. Data strongly suggest that PP1γ isoform of PP1 serine-threonine phosphatase, and not PP1α, participates in the pathway of regulation of insulin signaling and insulin resistance in neuronal cell.

### Effect of PP1α and PP1γ silencing on GLUT4 translocation and glucose uptake in insulin sensitive and insulin resistant neuronal cells

**Fig. 4:**
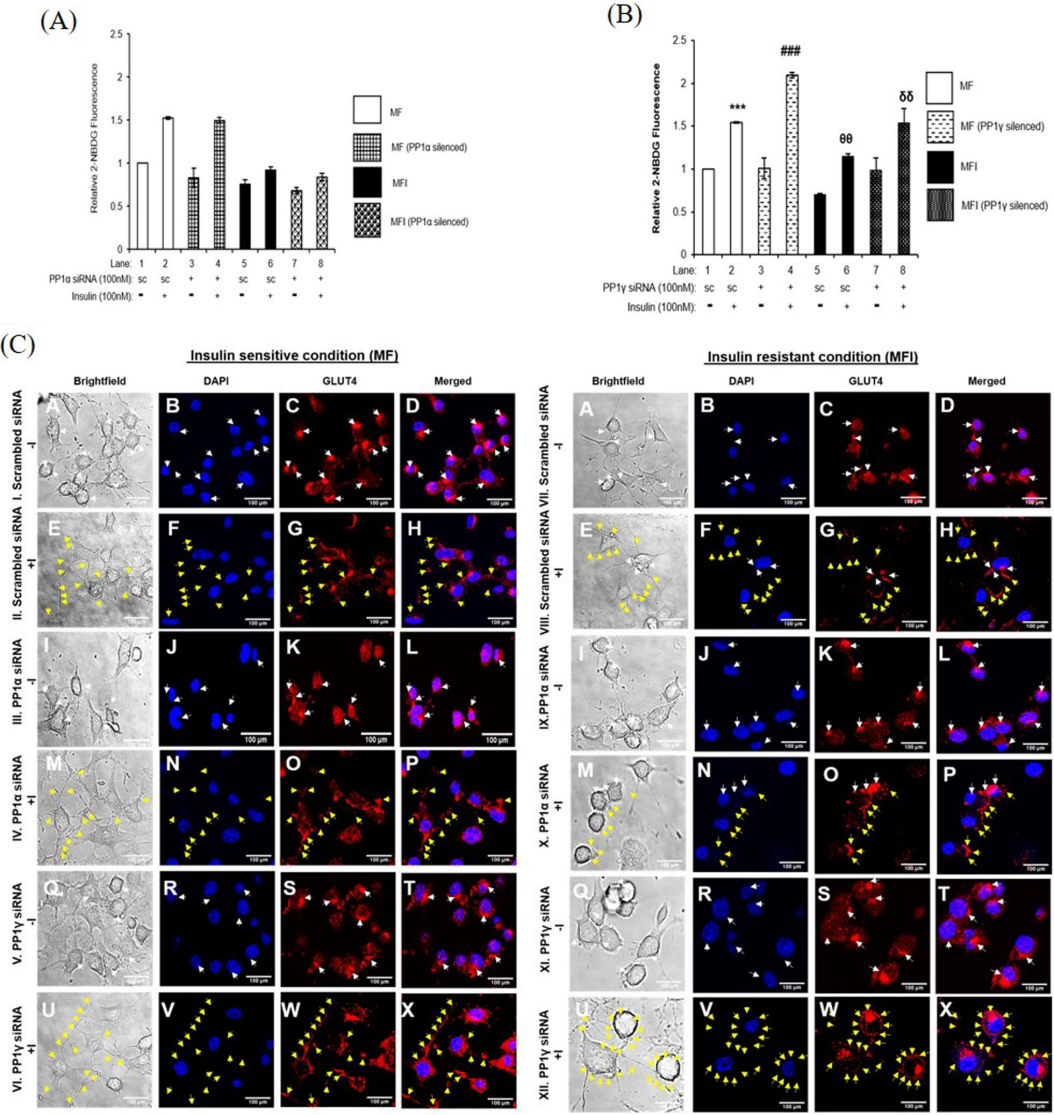
Effect of PP1α and PP1γ silencing on Glucose uptake and GLUT4 translocation in insulin sensitive and insulin resistant condition: Proliferated N2a cells were transfected with non-specific (scrambled) and PP1α and PP1γ specific siRNA. Post transfection cells were differentiated in the absence (MF; insulin sensitive) or chronic presence of 100 nM insulin (MFI; insulin resistant) for 3 days. **A** and **B** For glucose uptake assay, transfected N2a cells were serum-starved for 2 h and treated with 100 nM insulin for 30 min. Treated cells were lysed and uptake of 2-NBDG was then measured. Bar represents relative change in the uptake of 2-NBDG. Data expressed are mean ± SE. ***p < 0.001 compared to Lane 1, ^###^*p* < 0.001 compared to Lane 2, ^θθ^*p* < 0.01 compared to Lane 4 and ^δδ^*p* < 0.001 compared to Lane 6. Open bars: MF, filled bars: MFI, *A.U.* Arbitrary Units, *SC* Scrambled **C** For GLUT4 translocation, post transfection, MF MFI differentiated N2a cells were treated with or without 100 nM insulin for 30 min followed by fixation and permeabilization. Cells were subjected to immunofluorescence microscopy by using anti-goat Alexa 555 secondary antibody. Images were captured from different fields and a representative image of three images is presented. Scale Bar: 100 μm. White arrow: GLUT4 redistribution around the nucleus; Yellow arrows: GLUT4 redistribution/translocation on the plasma membrane

Looking at the participation in the insulin signaling pathway, we next tested the effect of PP1α and PP1γ silencing on neuronal GLUT4 translocation and glucose uptake. For glucose uptake assay, PP1α and PP1α silenced N2a cells were stimulated with or without 100 nM insulin for 30 min and subjected to 2 NBDG uptake [6, 7, 5, 20, 8–21]. PP1α silenced N2a cells did not show any change in uptake of glucose under insulin sensitive and insulin resistance (Fig. 4A). However, PP1γ silenced cells showed increase in insulin stimulated neuronal glucose uptake by 35 ± 0.03% (Fig. 4B, Lane 4 vs. Lane 2, ^###^*p* < 0.001) under insulin sensitive condition and 34 ± 0.17% under insulin resistant condition when compared to control (Fig. 4B, Lane 8 vs. Lane 6, ^δδ^*p* < 0.001). PP1α and PP1γ were also silenced in SH-SY5Y cells and stimulated with or without 100 nM insulin for 30 min and subjected to 2 NBDG uptake and we observed the similar trend (Additional file 1: Fig. S3). Data confirms that PP1γ isoform of PP1 serine-threonine phosphatase regulate neuronal insulin signaling and insulin resistance.

To validate and further confirm our observations, we tested GLUT4 translocation from inside the cell to the plasma membrane in PP1α and PP1γ gamma silenced N2a cells under insulin sensitive and insulin resistant condition, through confocal microscopy (Fig. 4C). In MF and MFI, under control (scrambled siRNA transfected) conditions, in absence of insulin, GLUT4 was localized at perinuclear region as well as dispersed in the cytoplasm (Fig. 4C, I and VII, Panel C, white arrows). In MF under control condition post insulin stimulation, GLUT4 was found to be present on the membrane (Fig. 4C, II, Panel G, yellow arrows). However, in MFI, under control condition post insulin stimulation, GLUT4 was found to be present on the membrane (Fig. 4C, VIII, Panel G, yellow arrows) (with a limited occurrence at the perinuclear region as well) (Fig. 4C, VIII, Panel G, white arrows). In MF and MFI under PP1α silenced condition in absence of insulin, GLUT4 was localized as similar to respective controls (Fig. 4C, III, IX, Panel K, white arrows). In MF, under PP1α silenced condition post insulin stimulation, GLUT4 was found onto the membrane (Fig. 4C, IV, Panel O, yellow arrows) similar to the respective control (Fig. 4C, II, Panel G vs. IV, Panel O). Similarly, in MFI, under PP1α silenced condition post insulin stimulation, GLUT4 was found on the membrane (Fig. 4C, X, Panel O, yellow arrows) similar to the respective control (Fig. 4C, VII, Panel G vs. X, Panel O). In MF, under PP1γ silenced condition post insulin stimulation, GLUT4 was found onto the membrane (Fig. 4C, VI, Panel W, yellow arrows) considerably more than the respective control (Fig. 4C, II, Panel G vs. VI, Panel W). In MFI, under PP1γ silenced condition post insulin stimulation, GLUT4 was found on the membrane (Fig. 4C, XII, Panel W, yellow arrows) considerably more than the respective control (Fig. 4C, VII, Panel G vs. XII, Panel W). This GLUT4 translocation (Fig. 4C) is in coherence with the glucose uptake (Fig. 4A and B), wherein only PP1γ silencing increases GLUT4 translocation and glucose uptake in neuronal cells but not PP1α silencing. Data revalidates and confirms that PP1γ regulates the neuronal insulin signaling and insulin resistance by dephosphorylating AKT2 via AKT-AS160-GLUT4 axis.

### Effect of silencing of AKT isoforms, PP1α and PP1γ on GSK3 isoforms in insulin sensitive and insulin resistant condition

Post PP1 inhibition, we observed increase in phosphorylation of GSK3β, while decrease in phosphorylation of GSK3α (Fig. 1G and H). We also observed PP1γ mediated specific dephosphorylation of AKT2 (Fig. 3E) and reduced neuronal glucose uptake (Fig. 4B). Therefore, we tested whether this decrease in phosphorylation of GSK3α could be the effect of differential regulation by AKT isoforms. We have previously reported isoform specific effect of AKT in regulating phosphorylation of AS160 by AKT isoform specific silencing [5]. In the current study we used the same strategy and methodologies to study AKT isoform specific role in regulating phosphorylation of GSK3α. Post AKT isoform specific silencing, differentiated N2A cells with or without insulin stimulation (100 nM, 30 min), were subjected to western immunoblotting probed with anti-phospho GSK3α, anti-phospho GSK3β, anti- GSK3α and anti- GSK3β antibodies.

**Fig. 5:**
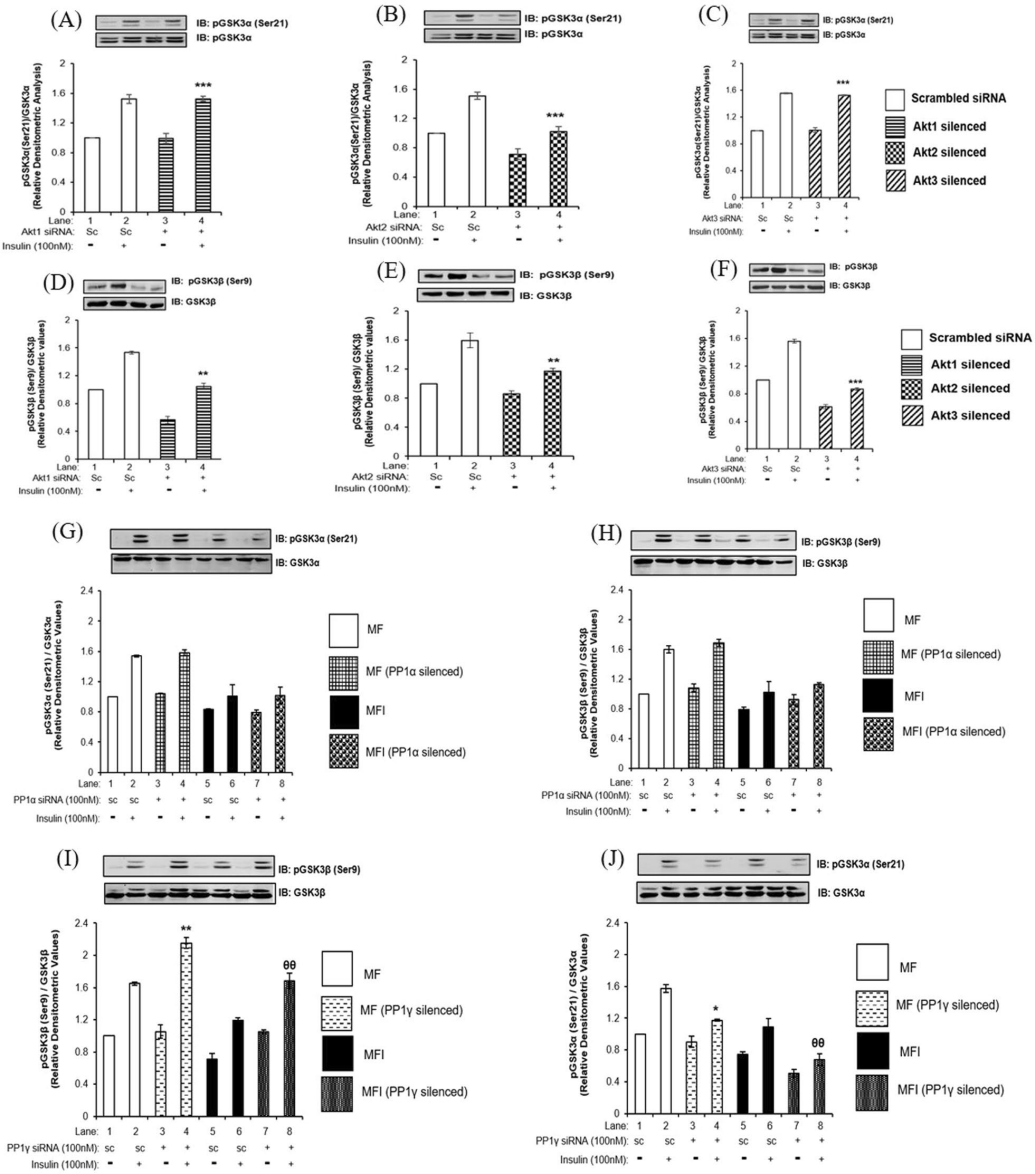
Effect of silencing of AKT isoforms, PP1α and PP1γ on GSK3 isoforms in insulin sensitive and insulin resistant condition: Three days post-proliferation, N2A cells were transfected with **A–F** AKT1, AKT2, and AKT3) specific siRNA and then differentiated in 2% DMSO for 3 days. Cells were stimulated with or without 100 nM insulin for 30 min prior to cell lysis. **G**–**J** Proliferated N2a cells were transfected with non-specific (scrambled) and PP1α and PP1γ specific siRNA. Post transfection cells were differentiated in the absence (MF; insulin sensitive) or chronic presence of 100 nM insulin (MFI; insulin resistant) for 3 days. Transfected N2a cells were treated with or without 100 nM insulin for 30 min. Lysates was subjected to western blotting, followed by probing with relevant primary antibodies. Bar represents relative change in **A**–**C**, **G** and **J** pGSK3α (Ser 21) when probed with anti-GSK3α antibody. **D**–**F**, **H** and **I** Bar represents relative change in pGSK3β (Ser9) when probed with anti-GSK3β antibody. Experiments were executed three times and a representative result is shown. Data expressed are mean ± SE. ***p < 0.001 as compared to Lane 1; ^###^*p* < 0.001, ^##^*p* < 0.01 as compared to Lane 2, ^θθ^*p* < 0.01 as compared to Lane 4. *IB* Immunoblot. Open bars: MF, filled bars: MFI, *IP* Immunoprecipitation *IB* Immunoblot, *SC* Scrambled

The phosphorylation of GSK3α (Ser21) was decreased by 32.54 ± 0.06% (Fig. 5B, Lane 4 vs. Lane 2, ***p < 0.001) post AKT2 silencing. However, there was no effect on phosphorylation of GSK3α (Ser21) post AKT1 and AKT3 silencing when compared to respective control (Fig. 5A and C). On the other hand, the phosphorylation of GSK3β at Ser9 was decreased by 35 ± 0.1% (Fig. 5D, Lane 4 vs. Lane 2, **p < 0.01) post AKT1 silencing, by 32 ± 0.05% (Fig. 5E Lane 4 vs. Lane 2, **p < 0.01) post AKT2 silencing and by 44 ± 0.01% (Fig. 5F, Lane 4 vs. Lane 2, ***p < 0.001) post AKT3 silencing when compared with their respective controls. Thus, AKT isoform specific silencing demonstrates that while all AKT isoforms regulate phosphorylation of GSK3β, only AKT2 regulates phosphorylation of GSK3α. Although this reports AKT isoform specific regulation of GSK3 isoforms in neuronal insulin signaling and resistance for the first time, this still does not answer the increase in phosphorylation of GSK3α post PP1 inhibition.

To determine whether PP1 isoforms regulate the phosphorylation of GSK3α, we tested the effect of PP1α and PP1γ silencing on phosphorylation of GSK3α at Ser21 and GSK3β at Ser9 in insulin sensitive and insulin resistant neuronal cells. PP1α silencing did not cause any change in phosphorylation of GSK3α at Ser21 (Fig. 5G) and GSK3β at Ser9 (Fig. 5H) in insulin sensitive and insulin resistant cells as compared to respective controls. However, PP1γ silencing showed 30 ± 0.07% increase in phosphorylation of GSK3β at Ser9 under insulin sensitive condition when compared to respective control (Fig. 5I, Lane 4 vs. Lane 2,**p < 0.01). Under insulin resistant condition, PP1γ silencing caused further 42 ± 0.09% increase in phosphorylation of GSK3β at Ser9 when compared to respective control (Fig. 5I, Lane 8 vs. Lane 6, ^θθ^*p* < 0.01). On the contrary, PP1γ silencing caused 25 ± 0.01% decrease in phosphorylation of GSK3α at Ser21 under insulin sensitive condition (Fig. 5J, Lane 4 vs. Lane 2, *p < 0.05) and further decrease by 38 ± 0.07% under insulin resistant condition (Fig. 5J, Lane 8 vs. Lane 6, ^θθ^*p* < 0.01) when compared to their respective controls.

Thus, PP1γ silencing (but not PP1α) increased phosphorylation of GSK3β and decreased phosphorylation of GSK3α in insulin sensitive and insulin resistant neuronal cells. This data corroborates with our PP1 inhibition data. Interestingly, what we observed here is that inhibition or silencing of a phosphatase resulted into decrease in phosphorylation of GSK3α. Further studies were required to address this issue.

### Effect of PP1α and PP1γ silencing on MLK3 and IKK in insulin sensitive and insulin resistant condition

To further investigate this issue, we ventured into possible intermediate proteins between the pathway of PP1 and GSK3α. Since GSK3α phosphorylation was getting affected, one of the possibilities was of a kinase, modulated under certain circumstances relaying PP1 specific effects downstream.

**Fig. 6:**
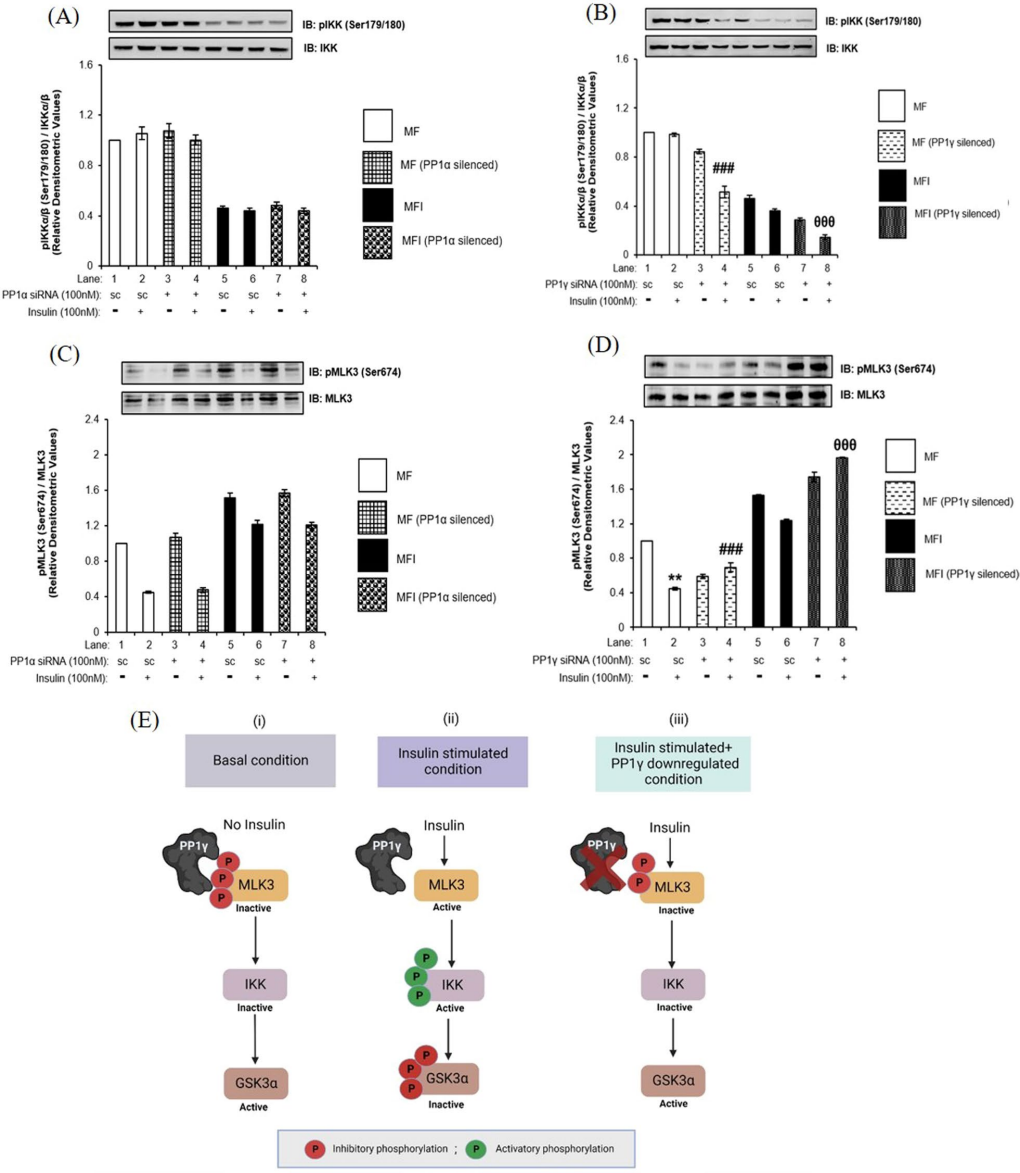
Effect of PP1α and PP1γ silencing on MLK3 and IKK in insulin sensitive and insulin resistant condition: **A**–**D** Proliferated N2a cells were transfected with non-specific (scrambled) and PP1α and PP1γ specific siRNA. Post transfection cells were differentiated in the absence (MF; insulin sensitive) or chronic presence of 100 nM insulin (MFI; insulin resistant) for 3 days. Transfected N2a cells were treated with or without 100 nM insulin for 30 min, lysed and probed with relevant primary antibodies for immunoblotting. Bar represents relative change in **A** and **B** pIKKα/β (Ser179/180) **C** and **D** pMLK3 (Ser674). Experiments were executed three times and a representative western blot is shown. Data expressed are mean ± SE. **p < 0.01, compared to Lane 1, ^###^*p* < 0.001 compared to Lane 2, ^θθθ^*p* < 0.001 compared to lane 4. **A** and **B** IKKα, **C** and **D** MLK3 was used as a loading control. Open bars: MF, filled bars: MFI, *IB* Immunoblot, *SC* Scrambled. (E) (i) Under basal condition, when PP1γ is present and insulin is not present, MLK3 is phosphorylated at its inhibitory site leading to its inactivation. Inactivated MLK3 cannot phosphorylate and activate IKK which in turn leads to the activation of GSK3α. (ii) Under insulin stimulated condition, when PP1γ is present it removes inhibitory phosphorylation of MLK3 and activating it. Activated MLK3 phosphorylates and activates IKK causing GSK3α phosphorylation and inactivation. (iii) When PP1γ was downregulated, it cannot dephosphorylate MLK3 causing its inactivation. Inactivated MLK3 in turn cannot phosphorylate and activate IKK leading to activation of GSK3α. Created with BioRender.com

Previously, two kinases, namely AKT2 and IKK, have been reported to specifically regulate phosphorylation of GSK3α in 3T3-L1 adipocytes and 293-IL-1R, respectively [23, 24]. However, in our study, we found that decrease in phosphorylation of GSK3α was not due to differential effect of AKT isoforms. This prompted us to investigate the possible role of IKK in regulating the phosphorylation of GSK3α. We tested the effect of PP1α and PP1γ silencing on the activity of IKK (as determined by phosphorylation at Ser179/180) under insulin sensitive and insulin resistant neuronal cells. PP1α silencing did not affect phosphorylation of IKK at Ser179/180 under both insulin sensitive and insulin resistant conditions (Fig. 6A). However, PP1γ silencing decreased phosphorylation of IKK by 48 ± 0.04% under insulin sensitive condition (Fig. 6B, Lane 4 vs. Lane 2, ^###^*p* < 0.01) and 60 ± 0.01% decrease under insulin resistant condition when compared to respective control (Fig. 6B, Lane 8 vs. Lane 4,^θθθ^*p* < 0.01). Thus, PP1γ specific effects in regulating GSK3α could be mediated by upstream kinase IKK and not AKT2 (as depicted in Fig. 6E). Since, decrease in IKK phosphorylation was observed post PP1γ silencing, this prompted us to look further upstream in the signaling cascade. Previously, MLK3 is one such kinase reported to negatively regulate IKK in NIH3T3 fibroblasts [25]. To test if PP1 regulates MLK3 and if this regulation extends to IKK and GSK3α, we tested the effect of PP1α and PP1γ silencing on phosphorylation of MLK3 at Ser674 post insulin stimulation under insulin sensitive and insulin resistant conditions. Under insulin sensitive control condition (scrambled siRNA transfected), post insulin stimulation the phosphorylation of MLK3 at Ser674 was decreased by 55 ± 0.009% (Fig. 6C and D, Lane 2 vs. Lane 1, ***p < 0.001). However, post insulin stimulation, PP1γ silencing led to 55 ± 0.05% (Fig. 6D, Lane 4 vs. Lane 2, ^###^*p* < 0.001) increase in phosphorylation of MLK3 under insulin sensitive condition, and 170 ± 0.04% % (Fig. 6D, Lane 8 vs. Lane 4, ^θθθ^*p* < 0.001) increase under insulin resistant condition, as compared to their respective controls. Interestingly, silencing of PP1α did not affect phosphorylation of MLK3 under all conditions tested.

Thus, under insulin sensitive and insulin resistant condition, PP1γ, and not PP1α, dephosphorylates and activates MLK3. Activated MLK3 phosphorylates its downstream kinase IKK which in turn phosphorylates GSK3α (as depicted in Fig. 6E (ii)). When PP1γ was silenced, it could not dephosphorylate MLK3, causing its inactivation (as depicted in Fig. 6E (iii)). Inactivated MLK3 could not phosphorylate and activate IKK leading to decreased GSK3α phosphorylation (as depicted in Fig. 6E (iii). Thus, GSK3α phosphorylation is mediated by PP1γ via ML3-IKK and not via AKT2 in neuronal cells (as depicted in Fig. 6E).

### Effect of MLK3 inhibition on activities of IKK and GSK3α

**Fig. 7:**
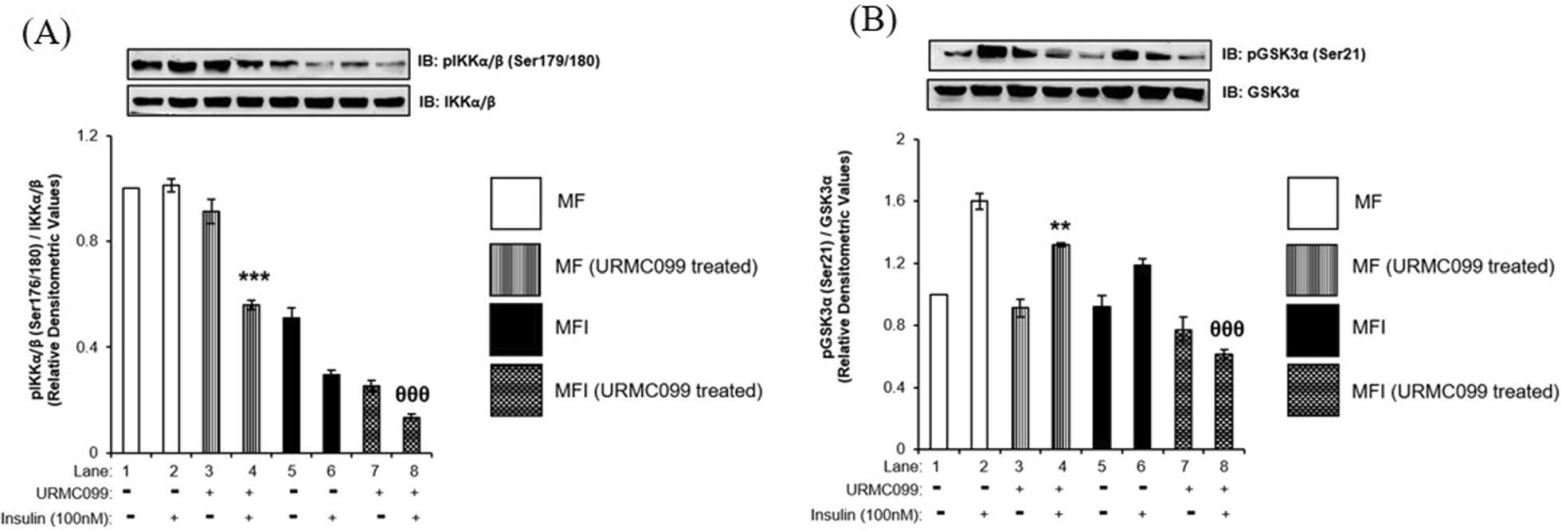
Effect of MLK3 inhibition on activities of IKK and GSK3α: N2a cells were differentiated in the absence (MF; insulin sensitive) or chronic presence of 100 nM insulin (MFI; insulin resistant) for 3 days and treated with or without 10 µM URMC099 for 2 h followed by 100 nM insulin for 30 min. Treated cells were lysed and subjected to western immunoblotting followed by probing with relevant primary antibodies. Bar represents relative change in **A** pIKKα/β (Ser179/180), **B** pGSK3α (Ser21). Experiments were executed three times and a representative western blot is shown. Data expressed are mean ± SE. ***p < 0.001 compared to Lane 2, ^θθθ^*p* < 0.001 compared to lane 4. **A** IKKα, **B** GSK3α was used as a loading control. Open bars: MF, filled bars: MFI, *IB* Immunoblot

To confirm that indeed PP1γ mediated dephosphorylation of MLK3 regulates the activity of GSK3α through IKK, we inhibited MLK3 by its specific inhibitor, URMC099 [26], and tested the effect of inhibition on the activity of IKK and GSK3α. We treated N2a cells with different concentrations of URMC and tested the effect on the phosphorylation of MLK3 at Ser674. Dose-dependent inactivation of MLK3 was observed with a maximum inhibition at 10 μM concentration of URMC (Additional file 1: Fig. S4). We therefore treated N2a cells with 10 μM URMC and tested the effect of inhibition on the activations of IKK and GSK3α. Both under insulin sensitive and insulin resistant conditions, the inhibition of MLK3 reduced the activation of IKK by 45 ± 0.03% and 55 ± 0.03% (Fig. 7A, Lane 4 vs. Lane 2 and Lane 8 vs. Lane 6, ***p < 0.01, ^θθθ^*p* < 0.01) and GSK3α by 17 ± 0.01% and 48 ± 0.03% (Fig. 7B, Lane 4 vs. Lane 2 and Lane 6 vs. Lane 8, ***p < 0.001, ^θθθ^*p* < 0.001) respectively, confirming the activation of GSK3α being regulated via MLK3-IKK arm.

### Effect of PP1α and PP1γ silencing on AD-like phenotypes

**Fig. 8:**
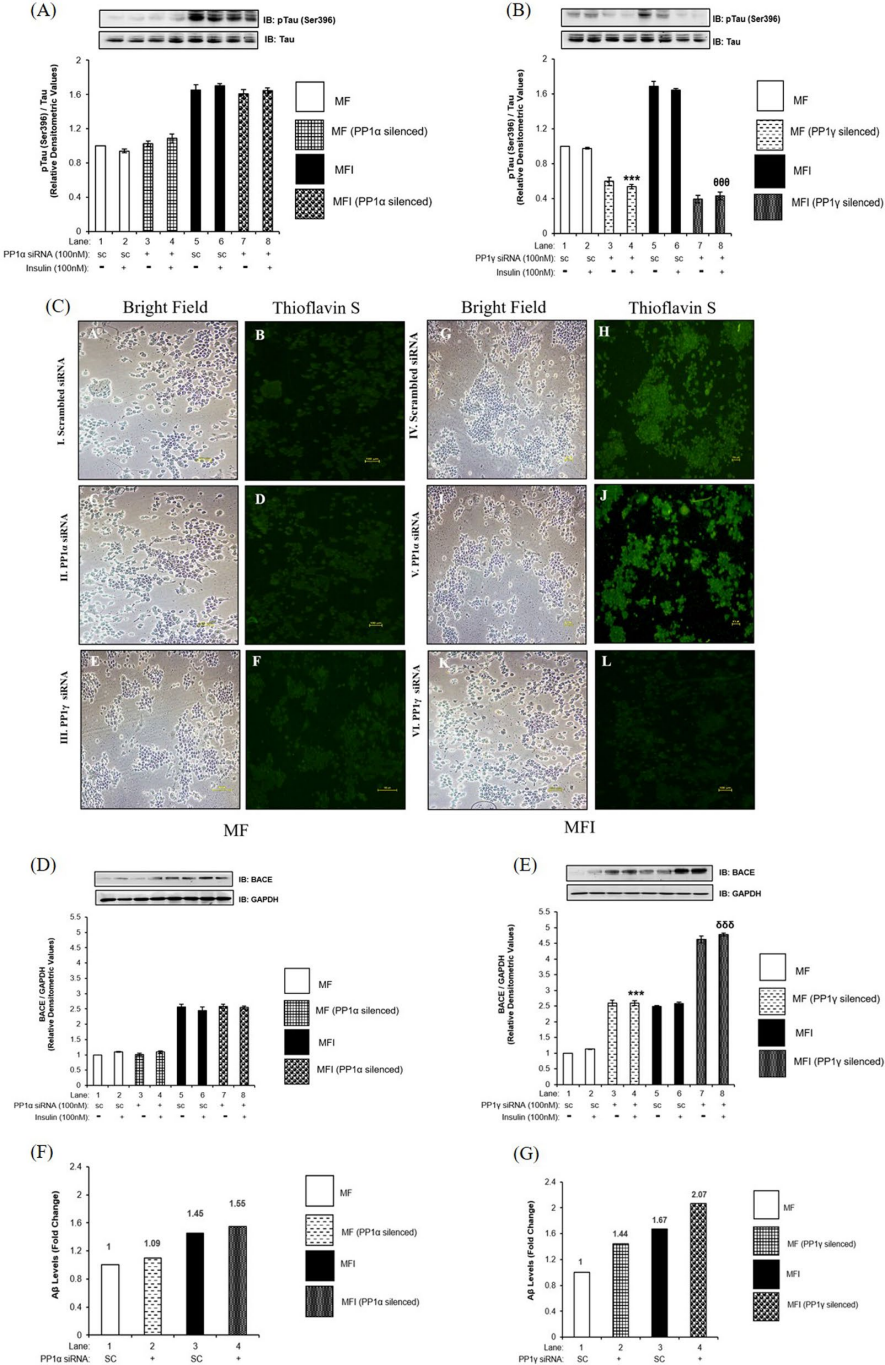
Effect of PP1α and PP1γ silencing on AD markers: (**A**, **B** and **D**, **E**) Proliferated N2a cells were transfected with non-specific (scrambled) and PP1α and PP1γ specific siRNA. Post transfection cells were differentiated in the absence (MF; insulin sensitive) or chronic presence of 100 nM insulin (MFI; insulin resistant) for 3 days. Transfected N2a cells were treated with or without 100 nM insulin for 30 min, lysed and probed with relevant primary antibodies for immunoblotting. Bar represents relative change in **A** and **B** pTau (Ser396), **C** and **D** BACE. Experiments were executed three times and a representative western blot is shown. Data expressed are mean ± SE. ***p < 0.001 compared to Lane 2, ^θ^*p* < 0.05 compared to lane 4, ^δδδ^*p* < 0.001 compared to Lane 6. **C** For thioflavin S staining proliferated N2a cells were transfected with non-specific (scrambled) and PP1α and PP1γ specific siRNA. Post transfection cells were differentiated in the absence (MF; insulin sensitive) or chronic presence of 100 nM insulin (MFI; insulin resistant) for 3 days. Transfected N2a cells were fixed, permeabilized, stained using ThS stain and visualized under an immunofluorescence microscope. Experiments were executed twice and representative images are shown. Scale bar: 100 μm. **F** and **G** For Amyloid-β measurement PP1α and PP1γ was silenced and conditioned media was collected, concentrated 2 times using speed vacuum and secreted amyloid-β (1–42) levels were measured using Beta-amyloid (1–42) colorimetric ELISA kit. Experiments were executed twice and average is shown. **A** and **B** Tau, **D** and **E** GAPDH was used as a loading control. Open bars: MF, filled bars: MFI, *IB* Immunoblot, *SC* Scrambled

**Fig. 9:**
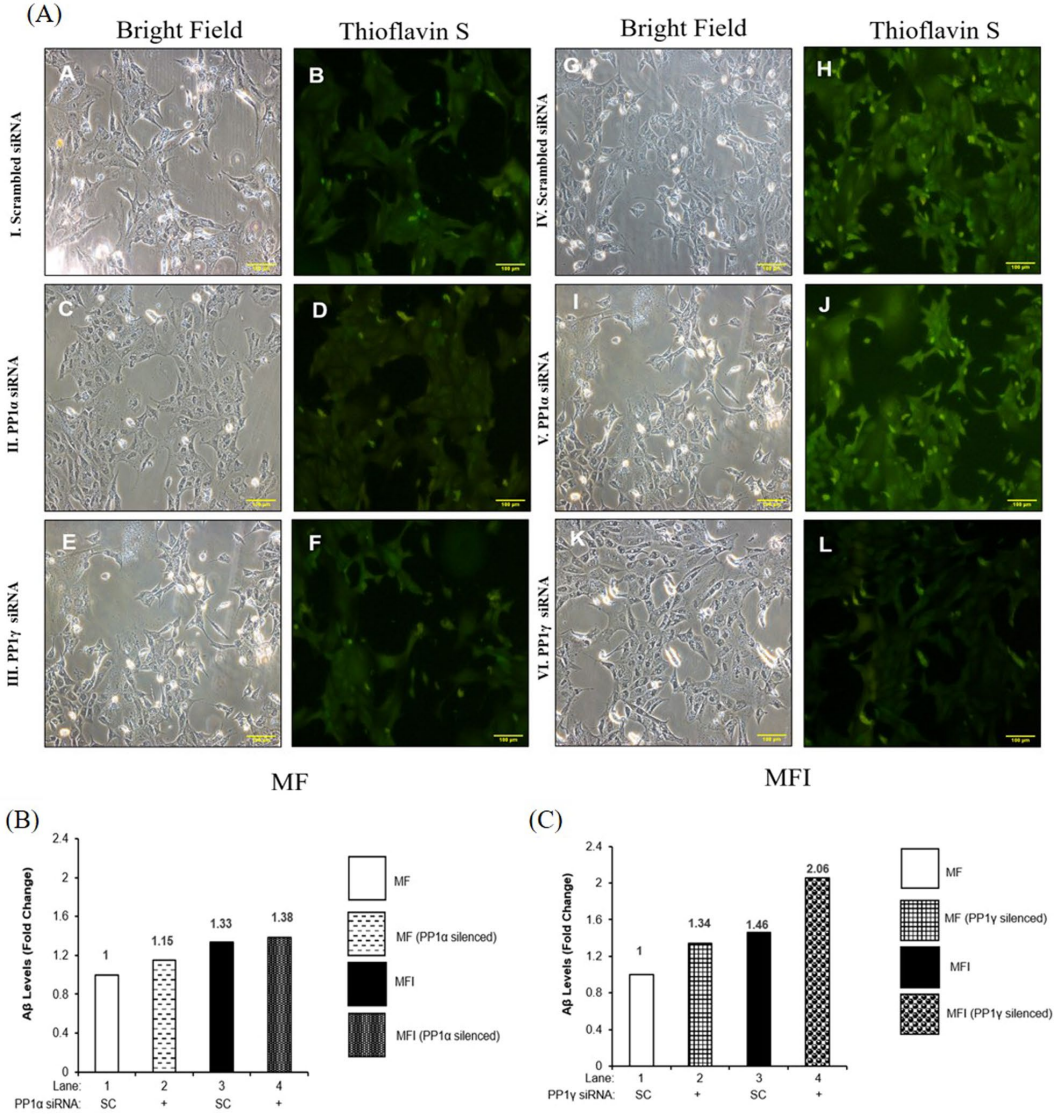
Effect of PP1α and PP1γ silencing on AD markers in SH-SY5Y cells: Proliferated N2a cells were transfected with non-specific (scrambled) and PP1α and PP1γ specific siRNA. Post transfection cells were differentiated in the absence (MF; insulin sensitive) or chronic presence of 100 nM insulin (MFI; insulin resistant) for 4 days. **A** For thioflavin S staining differentiated SH-SY5Y cells were fixed, permeabilized, stained using ThS stain and visualized under an immunofluorescence microscope. Experiments were executed twice and representative images are shown. Scale bar: 100 μm. **B** and **C** For Amyloid-β measurement PP1α and PP1γ was silenced and conditioned media was collected, concentrated 2 times using speed vacuum and secreted amyloid-β (1–42) levels were measured using Beta-amyloid (1–42) colorimetric ELISA kit. Experiments were executed twice and average is shown

Previous studies have already established connection between defective insulin signaling and involvement of GSK3 isoforms in the development of pathological condition, like AD [14–28]. Studies also established insulin resistance leading to AD [15, 14, 29, 30]. Our laboratory has previously reported role of a phosphatase, PTEN, in regulating neuronal insulin resistance and AD [31]. Therefore, in order to address the same, we wished to determine whether insulin resistance, further aggravated by PP1γ, caused AD-like phenomena. To achieve this, we tested the effect of PP1α and PP1γ silencing on molecules like BACE and Tau, very well-known proteins to be involved in progression of AD pathogenesis. We also tested the effect of PP1α and PP1γ downregulation on the formation of Aβ plaques and neurofibrillary tangles (NFTs), two pathological hallmarks of AD. We found that both under insulin sensitive and insulin resistant conditions, PP1α downregulation did not affect expression and phosphorylation of Tau at Ser396 when compared to the respective control (Fig. 8A). Post PP1γ silencing, Ser 396 phosphorylation of Tau displayed decrease by 20 ± 0.04% (Fig. 8B, Lane 4 vs. Lane 8, ^θ^*p* < 0.001) under insulin resistant condition when compared to the sensitive. PP1α downregulation did not affect formation of NFTs (Fig. 8C, II and V, Panel D and J), however, PP1γ downregulation reduced the formation of NFTs both in insulin sensitive and insulin resistant cells (Fig. 8C, III and VI, Panel F and L). On the contrary, upon PP1α downregulation while the expression of BACE remained unaffected both under insulin sensitive and insulin resistant conditions (Fig. 8D), PP1γ downregulation caused 130 ± 0.07% increase in expression of BACE under insulin sensitive condition (Fig. 8E, Lane 4 vs. Lane 2, ***p < 0.001) and 86 ± 0.04% increase under insulin resistance when compared to control (Fig. 8E, Lane 8 vs. Lane 6, ^δδδ^*p* < 0.001). Downstream to BACE, we observed that PP1α downregulation did not affect formation of Aβ plaques (Fig. 8F) whereas PP1γ downregulation increased Aβ plaque formation by 48% (Fig. 8G, Lane 2 vs. Lane 1) under insulin sensitive condition and 23% (Fig. 8G, Lane 4 vs. Lane 3) under insulin resistance when compared to control. We tested the same in insulin sensitive (MF) and insulin resistant (MFI) SH-SY5Y cells. Data obtained post silencing was similar to what was observed in N2a cells (Fig. 9). The results demonstrate insulin resistance, further aggravated by PP1γ, caused AD-like phenotype however, through a phenomenon of oppositely regulating phosphorylation of GSK3α and GSK3β isoforms wherein one promotes AD-like phenotypes while the other prevent it. Data shows that PP1γ, on one hand, regulates neuronal insulin signaling and insulin resistance via AKT2-AS160-GLUT4-glucose uptake arm and AD-like phenomena via AKT2-GSK3β-Tau-NFT arm. On the other hand, PP1γ regulates AD markers via MLK3-IKK-GSK3α-BACE-Aβ plaques formation arm. Therefore, PP1γ act like a possible linker between two disorders, insulin resistant diabetes and AD through AKT2 and MLK3, generating a possibility leading to Type-3 diabetes.

## Discussion

### PP1 and its isoforms in neuronal insulin signaling and insulin resistance

The activity of PP1 relies on its interaction with catalytic and regulatory subunits which determines its substrate specificity. Both catalytic and regulatory subunits have various isoforms. Isoforms of catalytic subunits were reported to be differentially expressed in different tissues. Silva et al. [13] in mice extracts of various tissues like brain, liver, skeletal muscle, kidney, small intestine, heart, lung, spleen, thymus, and testis determined the expression of different isoforms of PP1 catalytic subunits. These studies found that different isoforms have different expression level in different tissues. The expression of catalytic subunit α and δ was found in all the above-mentioned tissues with a limited expression in skeletal muscle. However, PP1γ expression level was the highest in brain tissue. These tissue specific differential expression of these isoforms suggests the significance of different isoforms of catalytic subunit of PP1 and possible tissue specific complexity in functions. Whatever little is known, the role(s) of each isoform of catalytic subunit in insulin signaling has been inconclusive. Corvera et al. [32] and Rondinone et al. [9] suggested the participation of a phosphatase responsible for GLUT4 transport in response to insulin in rat and human adipose tissue respectively. Although both of these studies had reported possible role of a phosphatases in insulin signaling, they were neither phosphatase nor catalytic subunit specific studies. Sharma et al. [12] reported α catalytic subunit of PP1 as a regulator of AS160 dephosphorylation in skeletal muscle, without affecting AKT. Geetah et al. [11] reported the combination of PP1δ catalytic subunit with regulatory subunit 12A as a new member in regulating skeletal muscle insulin signaling pathway. There is no study yet that defined the involvement specifically of PP1 in regulating insulin mediated GLUT4 transport neither in peripheral tissues (skeletal muscle, hepatocytes, cardiomyocytes etc.) nor in neurons and none of these studies are on insulin resistance. In the current study, we report with regard to expression and regulatory activity of PP1 catalytic subunits in normal as well as hyperinsulinemia mediated insulin resistant mouse (N2a), human (SH-SY5Y) neuroblastoma cell lines and High-fat-diet-fed mice whole brain lysates. The expression of PP1α and PP1γ did not change post insulin stimulation or under insulin resistant condition in neuronal cells; however, their functionality, did.

### PP1γ regulates neuronal glucose uptake through AKT2-AS160 axis

Several studies have reported PP1 to regulate dephosphorylation of pan-AKT in signaling cascades other than insulin signaling [33–35]. Jiang et al. [36] reported that liposomal C6 ceramide activated PP1, which in-turn dephosphorylated AKT1 in human melanoma cell lines. This is the only study so far which discussed the specific role of PP1 in regulating AKT2, in contrast to previous studies those reported pan-AKT findings. Our study determines isoform specific interactions between these two proteins in insulin signaling in any cellular system. We observe PP1γ but not PP1α regulating AKT2 specifically, without affecting AKT1 and AKT3. This isoform specific effect gets translated to the downstream target AS160, which in turn affects the translocation of GLUT4 from cytoplasm to plasma membrane leading to the uptake of glucose inside the cell. We, therefore, for the first time report the role of catalytic subunit PP1γ in regulating neuronal insulin signaling and insulin resistance through one of the isoforms of AKT.

### PP1γ regulates GSK3 isoforms via AKT2 and MLK3-PP1γ regulates GSK3 isoforms via AKT2 and MLK3

Our data on PP1 inhibition (Sect. “Effect of PP1 inhibition on neuronal insulin signaling”) surprisingly demonstrated different regulation of phosphorylation of two GSK3 isoforms. Previous evidences reported that the phosphorylation of GSK3α and GSK3β have been regulated through different kinases [24, 40]. Gulen *et. al* [37] reported IKK to phosphorylate GSK3α and not GSK3β. Our study, interestingly, reports that PP1γ regulates the phosphorylation of GSK3β through AKT2 but of GSK3α through IKK. Additionally, post PP1γ silencing we also observed a decrease in phosphorylation of IKK, that pointed to a possibility of presence of an upstream kinase relaying PP1γ specific IKK regulation. Previously Cole et al. [25] demonstrated MLK3 negatively regulates the activity of IKK that in human ovarian cancer epithelial cells and murine NIH-3T3 fibroblast cells. Our results demonstrated that PP1γ dephosphorylates and activates MLK3 leading to the activation of IKK, which in turn phosphorylates GSK3α (as depicted in Fig. 6E). So, we here show that PP1γ regulates phosphorylation of two GSK3 isoforms through two different axes; phosphorylation of GSK3β regulated by AKT2-GSK3β axis and phosphorylation of GSK3α regulated by MLK3-IKK-GSK3α axis.

### PP1γ regulates AD markers

As mentioned earlier, previous studies have established connection between aberrant insulin signaling and engagement of GSK3 isoforms in the development of AD [14–28]. Prediabetic conditions and insulin resistance has been confirmed to lead to AD [14, 15, 29, 30, 27, 38]. PTEN, a phosphatase, has previously been reported from our laboratory to participate in AD under the predisposition of insulin resistance [31].

It has been well established that GSK3 plays important role in regulating pathophysiology of AD. Amongst the pathological hallmarks of AD, the most prominent are deposition of Aβ plaques and formation of neurofibrillary tangles (NFTs). Aβ is generated by the sequential cleavage of β- amyloid precursor protein (APP) by β-site APP-cleaving enzyme 1 (BACE1) [39]. Formation of NFTs is the result of Tau hyperphosphorylation [40]. Both the hallmarks of AD are under the control of two isoforms of GSK3. While GSK3α regulates expression of BACE1, ultimately controlling Aβ plaque formation GSK3 regulates Tau hyperphosphorylation, ultimately controlling formation of NFTs. Previously, some phosphatases have been implicated in AD, regulating either the hallmarks of AD directly or by regulating GSK3 isoforms [18]. Silva et al. [41] first reported the role of PP1 in Aβ secretion in COS-1 cells. Subsequently, Sun et al. [42] and Liv et al. [43] reported the role of PP1 in Tau hyperphosphorylation in rat hippocampus and human brain respectively. However, Bennecib et al. [44] reported that PP1 regulates Tau phosphorylation via GSK3β in rat forebrain brain slices. Menendez et al. [45] also corroborated that PP1/GSK3β imbalance regulates AD progression. In our study we see PP1γ mediated opposite phosphorylation/dephosphorylation of two isoforms of GSK3 regulating the hallmarks of AD in neuronal cells.

Rebelo et al. [46] reported that PP1γ specifically binds to, and regulates Aβ aggregation in COS-7 cells, rat hippocampal and cortical primary neurons, and in adult rat hippocampus and cortex. Most recently, Combs et al. (2021) reported that both wildtype and mutant pathological Tau bind to PP1α and PP1γ, but only PP1γ silencing rescued mutant Tau effects in primary hippocampal neurons [47]. In the present study we determined how GSK3 isoforms relay PP1γ specific effects in AD progression. In the current study, PP1γ fine tunes AD-like phenotype in neuronal system, where on one hand the silencing of PP1γ increased the formation of Aβ plaques while on the other hand silencing of PP1γ decreased the formation of NFTs. These opposite effects in a cell may be attributed to PP1γ specific effects on GSK3 isoforms wherein PP1γ silencing increased the phosphorylation of GSK3β, inactivated it, and hence reduced Tau phosphorylation. This reduction in Tau phosphorylation resulted in lesser formation of NFTs. Oppositely, in the absence of PP1γ, GSK3α phosphorylation was regulated by MLK3 followed by IKK. Inactivation of IKK by MLK3 post PP1γ silencing further reduced the phosphorylation of GSK3α causing its activation. This GSK3α activation enhanced the expression of BACE which ultimately increased the deposition of Aβ plaques inside the cell. Overall, it seems that PP1γ is involved in the progression of insulin resistance and also regulates AD markers. This is the first evidence so far associating PP1γ with AD-like pathogenesis. In depth research is required to fully establish the role of PP1γ in the progression of AD pathogenesis under diabetic condition.

In summary, the study determined that PP1γ regulates neuronal insulin signaling and insulin resistance by dephosphorylating AKT2 and is also involved in regulating AD-like phenotypes through GSK3. The study primarily utilizes two different cell lines and partially utilizes animal models. In depth animal studies are needed to corroborate the finding. But the results obtained so far provided the first ever evidence of involvement of PP1γ in neuronal insulin resistance and AD-like phenotypes.

## Experimental procedures

### Materials

Minimum Essential Media (MEM), Dulbecco's Modified Eagle Medium (DMEM), Fetal Bovine Serum (FBS) were purchased from Gibco., MCDB 201 medium (Cat No. M6770-1L), nutrient mixture F-12 Ham (Cat No. N3520-1L), dimethyl sulfoxide (DMSO), triton X-100 (Cat No. X100-100ML), retinoic acid (Cat No. R2625-50MG), Okadaic Acid (Cat No. 78111-17-8), anti GAPDH antibody (Cat No. 9545), thioflavin S stain (Cat. No. T1892-25G) were purchased from Sigma-Aldrich Co. (St. Louis, MO, USA). Anti Tau antibody (Cat No. 32274), anti α-tubulin antibody (Cat No. 32293), anti-rabbit HRP antibody (Cat No. SC-2357) was purchased from Santa Cruz Biotechnology Inc. Anti phospho-AKT (Ser473) antibody (Cat No. 9271), anti phospho-AKT (Thr308) antibody (Cat No. 9275), anti AKT antibody (Cat No. 9272), anti phospho-AKT1 (Ser473) antibody (Cat No. 9018), anti AKT1 antibody (Cat No. 2938), anti phospho-AKT2 (Ser474) antibody (Cat No. 8599), anti AKT2 antibody (Cat No. 3063), anti phospho-AS160 (Ser588) antibody (Cat No. 8730), anti phospho-AS160 (Thr642) antibody (Cat No. 8881), anti AS160 antibody (Cat No. 2447), anti phospho-GSK3α (Ser21) antibody (Cat No. 9316), anti GSK3α/β antibody (Cat No. 5676), anti phospho-GSK3β (Ser9) antibody (Cat No. 9336), anti GSK3β antibody (Cat No. 9315), anti PP1α antibody (Cat No. 2582), anti phospho-IKKα/β (Ser179/180) antibody (Cat No. 2796), anti IKKα antibody (Cat No. 2682), anti MLK3 antibody (Cat No. 2817), anti BACE antibody (Cat No. 5606), anti-mouse HRP antibody (Cat No. 7076) were purchased from Cell Signaling Technology. URMC099 (Cat No. S7343) was purchased from Selleckchem, FK506 (Cat No. 342500-5MG) was purchased from Calbiochem. Anti AKT3 antibody (Cat No. PA-41700), anti phosho-MLK3 (Ser674) antibody (Cat No. PA5-40303), anti phospho-Tau (Ser396) antibody (Cat No. 44-752G), anti PP1γ antibody (Cat No. PA1-12382), Trypsin EDTA (Cat No. 25200056), 2-(*N*-[7-nitrobenz-2-oxa-1, 3-diazol-4-yl] amino)-2 deoxy glucose (2-NBDG) (Cat No. N13195), mouse beta-amyloid [1–42] colorimetric ELISA kit (Cat No. KMB3441) and human beta-amyloid [1–42] colorimetric ELISA kit (Cat No. KHB3441) were purchased from Thermo Fisher Scientific. Mouse PP1α (5ʹUAGCGACUAAACACAUCAAUU3ʹ) and PP1γ (5ʹGCGGUGAAGUUGAGGCUUAUU3ʹ) specific siRNA, human PP1α (5ʹUGGAUUGAUUGUACAGGAAUU3ʹ) and PP1γ (5ʹ GCGGUGAAGUUGAGGCUUAUU3ʹ) specific siRNAs were designed and synthesized by Qiagen, Germany.

### Cell culture

Mouse neuroblastoma cells (N2a) were cultured in MEM media supplemented with 10% FBS and human neuroblastoma cells (SH-SY5Y) were cultured in DMEM and Ham’s F12 media supplemented with 10% FBS containing antibiotics-penicillin 100 IU/ml and streptomycin 100 mg/ml, at 37 °C in 5% CO_2_. Post proliferation, N2a cells were differentiated in MEM supplemented with 1% FBS and 2% DMSO at 37 °C in 5% CO_2_ for 3 days. SH-SY5Y cells were differentiated in DMEM supplemented with 1% FBS and 10 μM retinoic acid for 4 days. Insulin resistance was generated as described previously in our laboratory [6, 7, 5–22]. Briefly, for creating insulin resistance in N2a cells, post proliferation, cells were subjected to equal mixture of serum free media (MCDB 201 medium and nutrient mixture F-12 Ham) and 2% DMSO in the absence (MF; insulin-sensitive) or in chronic presence of 100 nM insulin for 3 days (MFI; insulin-resistant). The media was replaced after every 12 h. For creating insulin resistance in SH-SY5Y cells, post proliferation cells were differentiated in equal mixture of serum free media (MCDB 201 medium and nutrient mixture F-12 Ham) and 10 μM retinoic acid for 4 days. The media was replaced after every 12 h. Differentiated N2a and SH-SY5Y cells were treated with 100 nM insulin for 30 min as reported previously in our laboratory [6, 7, 5–22].

### Preparation of mice brain lysates

Insulin resistance was generated in sixteen weeks old high-fat-diet (HFD) fed Swiss Albino male mice in the laboratory of Dr. Prosenjit Mondal, Indian Institute of Technology-Mandi, over ten weeks, and brain tissues of those mice, which were kind gifts from Dr. Mondal, Indian Institute of Technology-Mandi, were used for our experiments. Glucose tolerance test and insulin tolerance tests were performed; however, perfusion was not done before brain collection. Mice were divided into two groups: normal diet (ND) and high-fat-diet (HFD), each group containing three animals. Levels of triglyceride, cholesterol, serum glutamicoxaloacetic transaminase (SGOT), serum glutamic-pyruvic transaminase (SPGT), and fasting blood glucose were elevated by 48%, 29%, 50%, 53%, 40%, respectively in HFD mice as compared to ND mice. All experiments were performed following the guidelines prescribed by CPCSEA with the approval of the Internal Animal Ethics Committee, Visva-Bharati (IAEC/VB/2017/01). Mice brain were lysed in a homogenizer in lysis buffer (50 mM HEPES pH 7.4, 150 mM NaCl, 1.5 mM MgCl 2, 1 mM EGTA, 10 mM sodium pyrophosphate, 50 mM sodium fluoride, 50 mM β-glycerophosphate, 1 mM Na 3VO 4, 1% Triton X-100 supplemented with 2 mM PMSF, 10 μg/ml each of leupeptin and aprotinin) at 4 °C for 15 min. Brain tissues were homogenized as described previously [5]. Supernatants were collected and protein was estimated by Bicinchoninic Acid (BCA) method. An equal amount of protein was loaded and resolved on SDS-PAGE, followed by western immunoblotting.

### Inhibitor treatment

For inhibiting PP1, differentiated N2a and SH-SY5Y cells were treated with 4 μM okadaic acid for 120 min [19], followed by 100 nM insulin for 30 min at 37°C in 5% CO_2_. For inhibiting MLK3, differentiated N2a cells were treated with or without different concentrations of URMC099 (0.1, 5 and 10 μM) for 2 h, followed by 100 nM insulin at 37 °C in 5% CO_2_.

### Gene silencing by siRNA transfection

For silencing AKT1:5ʹ ATGCTGTTCAGAGACATTTA3ʹ; AKT2:5ʹAACATTTCTCTGTAGCAGAA3ʹ and AKT3:5ʹGATTGATAATATATAGGAGGA3ʹ mouse specific siRNA was used as described previously [5]. For silencing PP1α and PP1γ in N2a cells, mouse specific PP1α (5ʹUAGCGACUAAACACAUCAAUU3ʹ) and PP1γ (5ʹGCGGUGAAGUUGAGGCUUAUU3ʹ) siRNA respectively [48, 49] was designed and synthesized by Qiagen, Germany. Proliferated N2a cells were subjected to transient transfection with PP1α and PP1γ specific or non-specific scrambled siRNA using lipofectamine 2000 in Opti-MEM media. Transfected cells were incubated in Opti-MEM media for 6 h at 37°C in 5% CO_2_, after completion of 6 h, media was changed and cells were incubated in MEM media supplemented with 10% FBS overnight. Post 12 h, N2a cells were differentiated as described in “cell culture” section. For silencing PP1α and PP1γ in SH-SY5Y cells, human specific PP1α (5ʹUGGAUUGAUUGUACAGGAAUU3ʹ) and PP1γ (5ʹ GCGGUGAAGUUGAGGCUUAUU3ʹ) siRNA respectively [50] was designed and synthesized by Qiagen, Germany. Transfection in SH-SY5Y cells was performed similar to N2a cells.

### Immunoprecipitation

Because a specific phospho-Akt3 antibody was not available to measure 508 phosphorylation levels of Akt3, Akt3 protein was immunoprecipitated using Akt3 specific 509 antibody (Thermo, PA-41700), and probed with Anti-phospho-Akt (serine-473) (CST, Cat. No. 510 4058). Anti-IgG conformational antibody (CST, Cat No. 3678) was used to mask remaining IgG 511 bands. Cells were proliferated and differentiated as described above. 500 µg of protein lysate with 512 certain amount of primary antibody (as referred in datasheet of the manufacturer) was added and 513 incubated overnight at 4 °C in a microfuge rotator at 10rpm (Test tube Rotator, Tarsons, Cat. No. 514 3070). Next day, protein A/G beads were added for 4hrs, along with lysis buffer and protease 515 inhibitors. After 4 h of incubation, cells were centrifuged at 5000 rpm (Centrifuge, Sigma Cat. No.: 2-16KL) for 5 min. Supernatant was discarded and pellet was washed 3 times with lysis buffer along with protease inhibitors. Pellet was suspended in SDS-PAGE sample buffer and heated at 90 °C. The protein samples were resolved on SDS-PAGE.

### Cell lysis and immunoblotting

Treated N2a and SH-SY5Y cells were stimulated with or without 100 nM insulin for 30 min at 37°C in 5% CO_2._ Cells were then lysed in lysis buffer (50 mM HEPES, 150 mM NaCl, 1.5 mM MgCl_2_, 1 mM EGTA, 10 mM Na_4_P_2_O_7_, 50 mM NaF, 50 mM β-glycerophosphate, 1 mM Na_3_VO_4_ and 1% triton X-100) as described previously from our laboratory for 30 min at 4 °C [6, 7, 5–22]. Cells were scraped and homogenates were centrifuged at 16,000 x g for 15 min at 4 °C. Supernatant containing protein was collected was estimated by Bicinchoninic Acid (BCA) method [51]. An equal amount of protein was loaded and resolved on SDS-PAGE and western immunoblotting was performed using specific antibodies. Immunoblots were quantified by Quantity One 1-D analysis software (Bio-Rad Laboratories, Inc).

### Glucose uptake assay

Glucose uptake was performed as described previously from our laboratory [6, 7, 5–22]. Briefly, differentiated N2A and SH-SY5Y cells were washed and subjected to glucose starvation for 2 h. Treated cells were subjected to 50 μM 2-NBDG for 1 h and lysed in lysis buffer (20 mM Tris–HCl pH 7.4, 40 mM KCl, 1% sodium deoxycholate and 1% NP-40) for 15 min at 25 °C. Cells were then scraped and homogenates were centrifuged at 13,000×*g* for 20 min at 4 °C. Supernatants were collected and fluorescence was measured using a fluorescence spectrophotometer LS55 (Perkin Elmer, USA) at the excitation and emission wavelengths of 485 nm and 535 nm, respectively.

### Confocal microscopy

Confocal microscopy of N2A cells was performed as described previously (Sharma and Dey in Cell Mol Life Sci 78:7873–7898, 2021;Bisht and Dey in BMC Cell Biol 9:48, 2008;). Briefly, proliferated N2a were transfected with scrambled and PP1α and PP1γ specific siRNA. Post transfection cells were differentiated in MF MFI medium followed by treatment with 100 nM insulin for 30 min. Treated cells were fixed with 4% paraformaldehyde for 15 min. Cells were permeabilized by incubating with 0.5% Triton X-100 for 5 min, followed by washing with 1X PBS. Cells were blocked in blocking buffer (BSA 1% in PBS) for 1 hour. Cells were incubated overnight with anti-GLUT4 antibody at 4 °C. Cells were washed using 1X PBS and incubated with Donkey anti-Goat Alexa 555 secondary antibody for 45 min at room temperature. Cells were washed further with 1X PBS and mounted using ProLong™ Diamond Antifade Mountant with DAPI on to glass slides. Images were taken using Leica TCS SP8 microscope using 63 X (with oil) confocal objective. Images were taken at Central Research Facility, Sonipat Campus, Indian Institute of Technology-Delhi, Haryana. Images were processed using Leica Application Suite X software.

### Thioflavin S staining

Thioflavin S staining was performed as described previously from our laboratory [53]. Briefly, N2a cells were grown on the coverslip and proliferated cells were transfected with PP2Cα specific siRNA as described above. Post transfection cells were differentiated in insulin-sensitive (MF) and insulin-resistant (MFI) conditions for 3 days as described above. On third day of differentiation coverslips was washed with 1X PBS and fixed with 3% paraformaldehyde for 15 min at room temperature (RT). Cells were permeabilized with 0.02% Triton X-100 for 10 min and treated with 0.01% Thioflavin-S (ThS) for 5 min at RT in dark. Excess stain was removed by washing cells with 70% ethanol and washed cells were kept in high phosphate buffer (mM: NaCl 411, KCl 8.1, Na_2_HPO_4_ 30, KH_2_PO_4_ 5.2, pH 7.2) for 30 min on ice. Cells were visualized under Nikon Eclipse Ti-S immunofluorescence microscope.

### Amyloid-β (1–42) measurement

Amyloid-β measurement was performed using Beta-amyloid (1–42) colorimetric ELISA kit (Invitrogen Corp., Camarillo, CA) as per manufacturer’s instructions [53]. Briefly, PP2Cα was silenced in N2a cells and subjected to differentiation and on the third day of differentiation conditioned media was collected and 1 mM 4-benzenesulfonyl fluoride hydrochloride (AEBSF) was added at RT. Collected media was then concentrated 4 times using Speed-vacuum and 100 µl of concentrated media was used for the assay.

### Statistical analysis

The data expressed as mean±SE. All the experiments were executed three times and a representative result was shown. All the data were analyzed using a two-tailed unpaired *t-*test. *p*<0.05 was considered statistically significant.

## Supplementary Information

**Additional file 1. Fig S1 Effect of PP1 inhibition on neuronal insulin signaling in SH-SY5Y cells:** Differentiated SH-SY5Y cells were treated with or without 4 μM OA for 120 min, followed by 100 nM insulin for 30 min. Treated cells were lysed and subjected to western immunoblotting followed by probing with relevant primary antibodies. Bar represents relative change in (A) pAKT (Ser473) (B) pAKT (Thr308) (C) pAS160 (Ser588) (D) pAS160 (Thr642) (F) pGSK3α (Ser21) (G) pGSK3β (Ser9). For glucose uptake assay differentiated N2a cells were serum-starved for 2 h and then treated with or without 4 μM OA for 120 min, followed by 100 nM insulin for 30 min. Uptake of 2-NBDG was then measured. Bar represents (E) relative change in the uptake of 2-NBDG. Experiments were executed three times and a representative western blot is shown. Data expressed are mean ± SE ***p < 0.001 compared to Lane 1. (A and B) AKT was used as a loading control (C and D) AS160 was used as a loading control (F) pGSK3α was used as a loading control (G) pGSK3β was used as a loading control. A.U.: Arbitrary Units. IB: Immunoblot, OA: Okadaic Acid. **Fig S2 Dose course of PP1α and PP1γ silencing in N2a cells:** Proliferated N2a cells were transfected with non-specific (scrambled) and different concentrations of PP1α and PP1γ specific siRNA. Post transfection cells were differentiated for 3 days, lysed and probed with relevant primary antibodies for immunoblotting. Bar represents relative change in expression of (A) PP1α and (B) PP1γ. Experiments were executed two times and a representative western blot is shown. Data expressed are average. α-Tubulin was used as a loading control. IB: Immunoblot, SC: Scrambled. **Fig S3 Effect of PP1α and PP1γ silencing on Glucose uptake in SH-SY5Y cells:** Proliferated SH-SY5Y cells were transfected with non-specific (scrambled) and PP1α and PP1γ specific siRNA. Post transfection cells were differentiated in the absence (MF; insulin sensitive) or chronic presence of 100 nM insulin (MFI; insulin resistant) for 4 days. (A and B) For glucose uptake assay, transfected SHSY5Y cells were serum-starved for 2 h and treated with 100 nM insulin for 30 min. Treated cells were lysed and uptake of 2-NBDG was then measured. Bar represents relative change in the uptake of 2-NBDG. Data expressed are mean ± SE. ***p < 0.001 compared to Lane 1, ^###^p < 0.001 compared to Lane 2, ^θθθ^p < 0.01 compared to Lane 4 and ^δδδ^p < 0.001 compared to Lane 6. Open bars: MF, filled bars: MFI, A.U.: Arbitrary Units, SC: Scrambled. **Fig S4 Dose course of URMC099 for MLK3 inhibition in N2a cells:** Differentiated N2a cells were treated with or without different concentrations of URMC099 for 2 h, followed by 100 nM insulin for 30 min. Treated cells were lysed and subjected to western immunoblotting followed by probing with pMLK3 (Ser674) and MLK3 antibodies. Bar represents relative change in pMLK3 (Ser473). Experiments were executed two times and a representative western blot is shown. Data expressed are average. MLK3 was used as a loading control. IB: Immunoblot.

## Data Availability

All data generated or analyzed during this study are included in this published article (and its supplementary information files).
