# Supplementary material for: PP1γ regulates neuronal insulin signaling and aggravates insulin resistance leading to AD-like phenotypes
Source: Cell Commun Signal. 2023 Apr 21;21:82. doi: 10.1186/s12964-023-01071-x (PMC10120118; doi:10.1186/s12964-023-01071-x)
Supplement: Supplementary file 2 — Additional file 1. Fig S1 Effect of PP1 inhibition on neuronal insulin signaling in SH-SY5Y cells: Differentiated SH-SY5Y cells were treated with or without 4 μM OA for 120 min, followed by 100 nM insulin for 30 min. Treated cells were lysed and subjected to western immunoblotting followed by probing with relevant primary antibodies. Bar represents relative change in (A) pAKT (Ser473) (B) pAKT (Thr308) (C) pAS160 (Ser588) (D) pAS160 (Thr642) (F) pGSK3α (Ser21) (G) pGSK3β (Ser9). For glucose uptake assay differentiated N2a cells were serum-starved for 2 h and then treated with or without 4 μM OA for 120 min, followed by 100 nM insulin for 30 min. Uptake of 2-NBDG was then measured. Bar represents (E) relative change in the uptake of 2-NBDG. Experiments were executed three times and a representative western blot is shown. Data expressed are mean ± SE ***p < 0.001 compared to Lane 1. (A and B) AKT was used as a loading control (C and D) AS160 was used as a loading control (F) pGSK3α was used as a loading control (G) pGSK3β was used as a loading control. A.U.: Arbitrary Units. IB: Immunoblot, OA: Okadaic Acid. Fig S2 Dose course of PP1α and PP1γ silencing in N2a cells: Proliferated N2a cells were transfected with non-specific (scrambled) and different concentrations of PP1α and PP1γ specific siRNA. Post transfection cells were differentiated for 3 days, lysed and probed with relevant primary antibodies for immunoblotting. Bar represents relative change in expression of (A) PP1α and (B) PP1γ. Experiments were executed two times and a representative western blot is shown. Data expressed are average. α-Tubulin was used as a loading control. IB: Immunoblot, SC: Scrambled. Fig S3 Effect of PP1α and PP1γ silencing on Glucose uptake in SH-SY5Y cells: Proliferated SH-SY5Y cells were transfected with non-specific (scrambled) and PP1α and PP1γ specific siRNA. Post transfection cells were differentiated in the absence (MF; insulin sensitive) or chronic presence of 100 nM insulin ( [file 12964_2023_1071_MOESM2_ESM.docx]

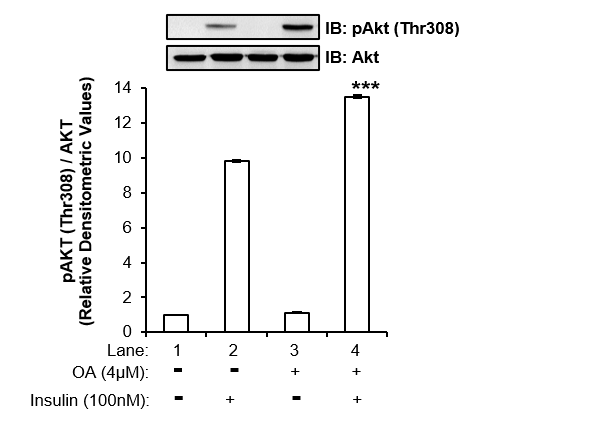

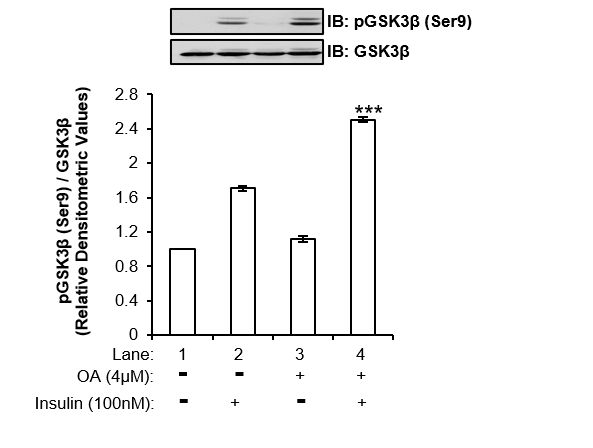

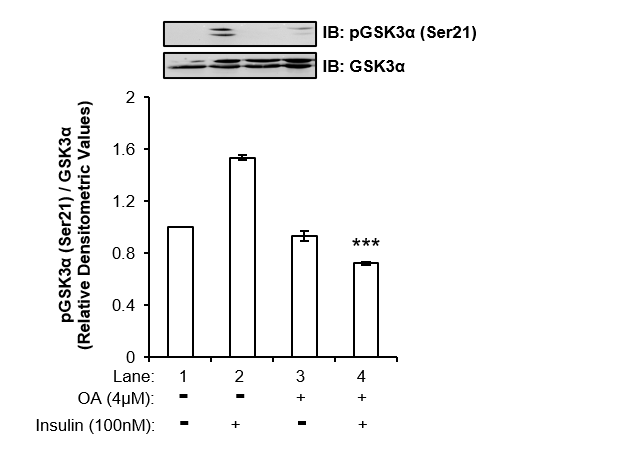

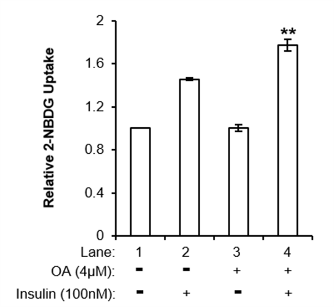

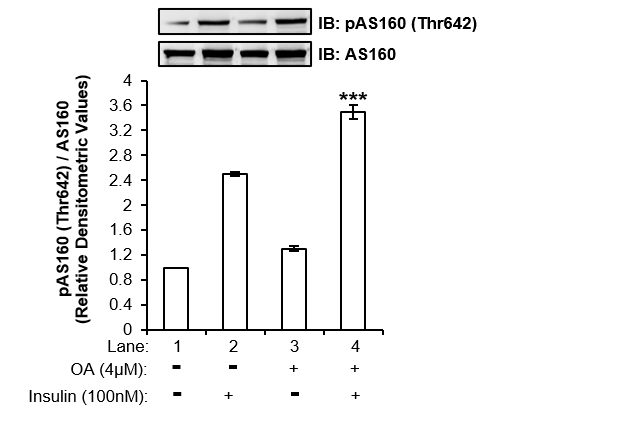

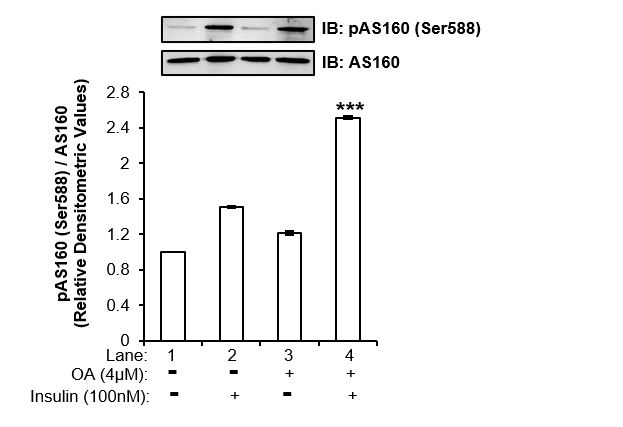

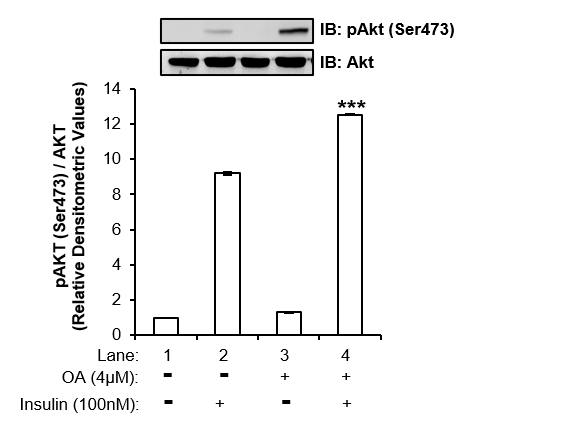


**Figure S1**

**Supplementary Information**

(E)

(G)

(F)

(D)

(C)

(B)

(A)

**Fig S1 Effect of PP1 inhibition on neuronal insulin signaling in SH-SY5Y cells:** Differentiated SH-SY5Y cells were treated with or without 4 μM OA for 120 min, followed by 100 nM insulin for 30 min. Treated cells were lysed and subjected to western immunoblotting followed by probing with relevant primary antibodies. Bar represents relative change in (A) pAKT (Ser473) (B) pAKT (Thr308) (C) pAS160 (Ser588) (D) pAS160 (Thr642) (F) pGSK3α (Ser21) (G) pGSK3β (Ser9). For glucose uptake assay differentiated N2a cells were serum-starved for 2 h and then treated with or without 4 μM OA for 120 min, followed by 100 nM insulin for 30 min. Uptake of 2-NBDG was then measured. Bar represents (E) relative change in the uptake of 2-NBDG. Experiments were executed three times and a representative western blot is shown. Data expressed are mean ± SE ***p<0.001 compared to Lane 1. (A and B) AKT was used as a loading control (C and D) AS160 was used as a loading control (F) pGSK3α was used as a loading control (G) pGSK3β was used as a loading control. A.U.: Arbitrary Units. IB: Immunoblot, OA: Okadaic Acid.


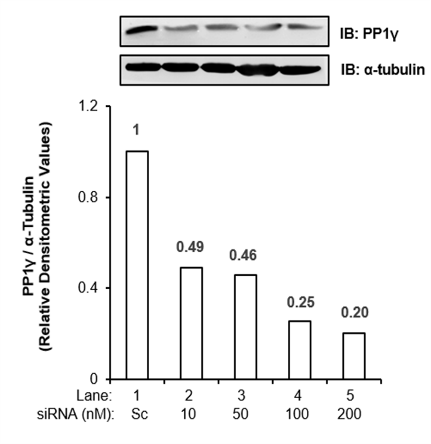

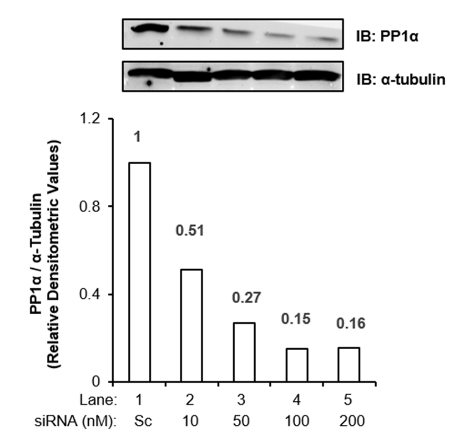


**Figure S2**

(B)

(A)

**Fig S2 Dose course of PP1α and PP1γ silencing in N2a cells:** Proliferated N2a cells were transfected with non-specific (scrambled) and different concentrations of PP1α and PP1γ specific siRNA. Post transfection cells were differentiated for 3 days, lysed and probed with relevant primary antibodies for immunoblotting. Bar represents relative change in expression of (A) PP1α and (B) PP1γ. Experiments were executed two times and a representative western blot is shown. Data expressed are average. α-Tubulin was used as a loading control. IB: Immunoblot, SC: Scrambled.


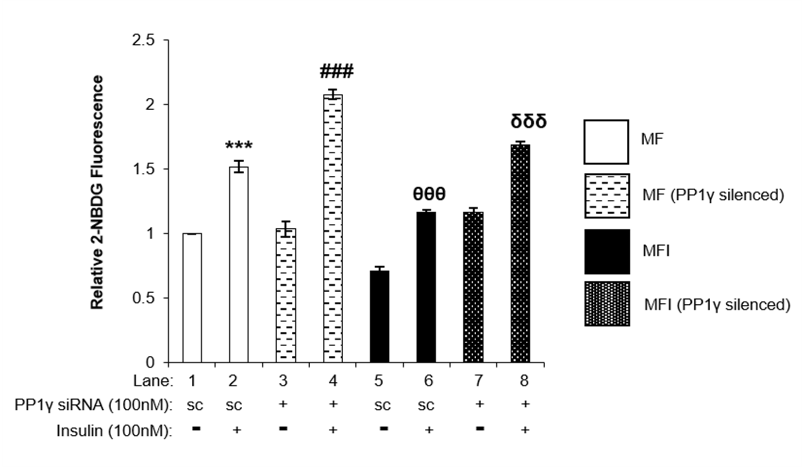

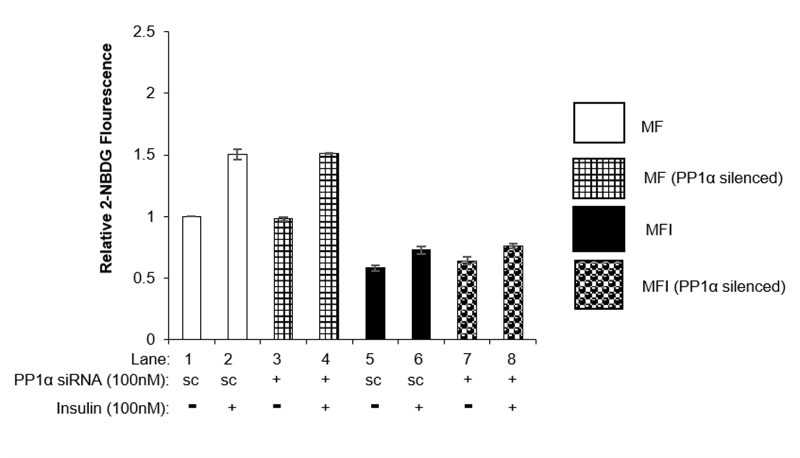


**Figure S3**

(A)

(B)

**Fig S3 Effect of PP1α and PP1γ silencing on Glucose uptake in SH-SY5Y cells:** Proliferated SH-SY5Y cells were transfected with non-specific (scrambled) and PP1α and PP1γ specific siRNA. Post transfection cells were differentiated in the absence (MF; insulin sensitive) or chronic presence of 100 nM insulin (MFI; insulin resistant) for 4 days. (A and B) For glucose uptake assay, transfected SHSY5Y cells were serum-starved for 2 h and treated with 100 nM insulin for 30 min. Treated cells were lysed and uptake of 2-NBDG was then measured. Bar represents relative change in the uptake of 2-NBDG. Data expressed are mean ± SE. ***p<0.001 compared to Lane 1, ^###^p<0.001 compared to Lane 2, ^θθθ^p<0.01 compared to Lane 4 and ^δδδ^p<0.001 compared to Lane 6. Open bars: MF, filled bars: MFI, A.U.: Arbitrary Units, SC: Scrambled.


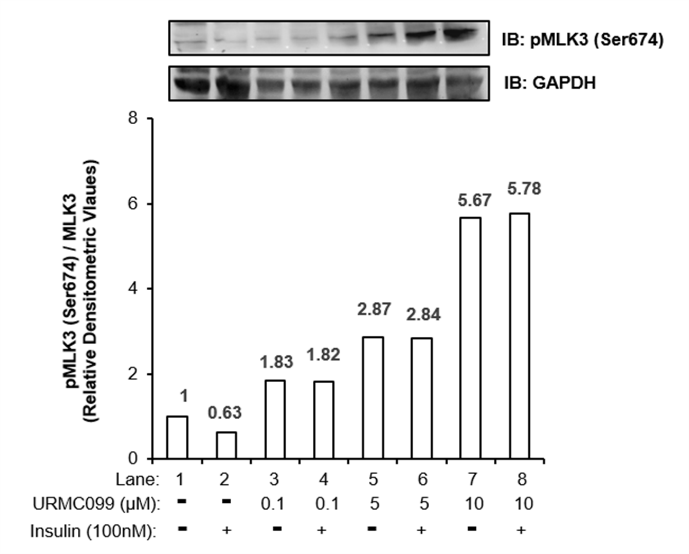


**Figure S4**

**Fig S4 Dose course of URMC099 for MLK3 inhibition in N2a cells:** Differentiated N2a cells were treated with or without different concentrations of URMC099 for 2 h, followed by 100 nM insulin for 30 min. Treated cells were lysed and subjected to western immunoblotting followed by probing with pMLK3 (Ser674) and MLK3 antibodies. Bar represents relative change in pMLK3 (Ser473). Experiments were executed two times and a representative western blot is shown. Data expressed are average. MLK3 was used as a loading control. IB: Immunoblot.
